# Systems-wide analysis of BCR signalosomes and downstream phosphorylation and ubiquitylation

**DOI:** 10.15252/msb.20145880

**Published:** 2015-06-02

**Authors:** Shankha Satpathy, Sebastian A Wagner, Petra Beli, Rajat Gupta, Trine A Kristiansen, Dessislava Malinova, Chiara Francavilla, Pavel Tolar, Gail A Bishop, Bruce S Hostager, Chunaram Choudhary

**Affiliations:** 1 Department of Proteomics, The Novo Nordisk Foundation Center for Protein Research, Faculty of Health and Medical Sciences, University of CopenhagenCopenhagen, Denmark; 2 Division of Immune Cell Biology, MRC National Institute for Medical ResearchMill Hill, London, UK; 3 Department of Microbiology, Graduate Program in Immunology and Department of Internal Medicine, University of IowaIowa City, IA, USA; 4 VAMCIowa City, IA, USA; 5 Department of Pediatrics, University of IowaIowa City, IA, USA

**Keywords:** BCL10, BCR, phosphorylation, RAB7A, ubiquitylation

## Abstract

B-cell receptor (BCR) signaling is essential for the development and function of B cells; however, the spectrum of proteins involved in BCR signaling is not fully known. Here we used quantitative mass spectrometry-based proteomics to monitor the dynamics of BCR signaling complexes (signalosomes) and to investigate the dynamics of downstream phosphorylation and ubiquitylation signaling. We identify most of the previously known components of BCR signaling, as well as many proteins that have not yet been implicated in this system. BCR activation leads to rapid tyrosine phosphorylation and ubiquitylation of the receptor-proximal signaling components, many of which are co-regulated by both the modifications. We illustrate the power of multilayered proteomic analyses for discovering novel BCR signaling components by demonstrating that BCR-induced phosphorylation of RAB7A at S72 prevents its association with effector proteins and with endo-lysosomal compartments. In addition, we show that BCL10 is modified by LUBAC-mediated linear ubiquitylation, and demonstrate an important function of LUBAC in BCR-induced NF-κB signaling. Our results offer a global and integrated view of BCR signaling, and the provided datasets can serve as a valuable resource for further understanding BCR signaling networks.

## Introduction

B lymphocytes play crucial roles in the adaptive immune system by recognizing foreign antigens and eliciting appropriate host protective responses. The developmental fate of B cells, as well as their functions in immune responses, is critically regulated by B-cell receptors (BCRs) (Rickert, 2013). Antigen-mediated cross-linking of BCRs leads to the assembly of the BCR signaling complexes (signalosomes), which generate downstream signaling outputs. The cellular response to BCR engagement depends on the activation of posttranslational modification (PTM)-based signaling networks. Phosphorylation has been identified as a key mediator of BCR signaling, and it is implicated in regulating diverse processes in B lymphocytes (Rickert, 2013; Mowen & David, 2014). Similarly, ubiquitylation plays critical roles in BCR signaling, most notably in the activation of the nuclear factor-kappaB (NF-κB) pathway (Thome *et al*, 2010).

Importantly, mutations in BCR signaling components can lead to uncontrolled activation of signaling, and dysregulation of BCR signaling has been implicated in the development of lymphoid malignancies and autoimmune disorders (Hasler & Zouali, 2001; Young & Staudt, 2013). Therefore, elucidating the details of BCR signaling is relevant for understanding its roles both in normal immune responses and in B-cell malignancies. While focused biochemical and genetic studies have identified important roles of phosphorylation and ubiquitylation in this system, the extent of their involvement in BCR signaling is not fully known.

Advances in quantitative mass spectrometry (MS)-based proteomics have enabled systems-wide analyses of signaling networks (Choudhary & Mann, 2010; Bensimon *et al*, 2012). This approach has been used to monitor the dynamics of thousands of phosphorylation sites in a single experiment (Olsen *et al*, 2006; Sharma *et al*, 2014). Recently, we and others have reported a streamlined proteomic method for the profiling of ubiquitin-derived di-glycine (di-Gly) remnants (Kim *et al*, 2011; Wagner *et al*, 2011), permitting large-scale, site-specific, and quantitative analysis of ubiquitylation in response to cellular perturbations (Povlsen *et al*, 2012; Iesmantavicius *et al*, 2014).

In this study, we combined multiple proteomic approaches to obtain a detailed picture of BCR signalosomes, and downstream phosphorylation and ubiquitylation signaling. We identified most of the known components of BCR signaling and implicate many additional proteins and PTMs in this system. We validated ANKRD13A as a novel constituent of BCR signalosome and showed that BCR-induced phosphorylation of RAB7A at S72 regulates its association with its effector proteins and with endo-lysosomal compartments. Additionally, we identified BCL10 as a *bona fide* target of BCR-induced linear ubiquitylation and demonstrated an important role of the linear ubiquitin ligase HOIP in BCR-induced phosphorylation of IκB. Together, these results expand the knowledge about the composition of BCR signalosomes and provide a systems-wide view of the downstream signaling.

## Results

### Strategy for analysis of BCR-regulated signaling networks

**Figure 1:**
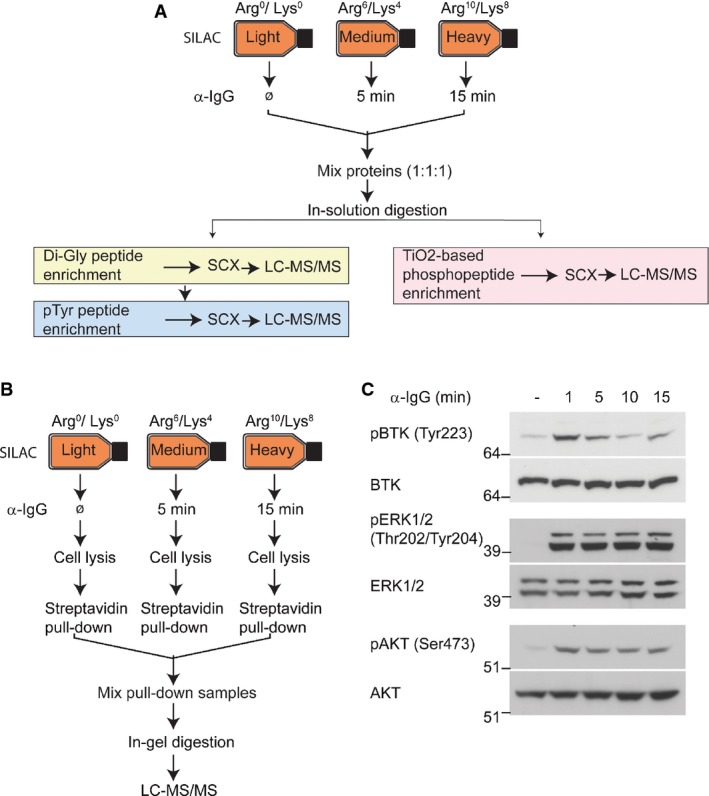
Strategy for proteomic analysis of BCR signaling

To obtain a multifaceted view of BCR signaling, we used MS-based proteomics to: (i) identify the components of BCR signalosome, (ii) quantify BCR-regulated phosphorylation events, and (iii) monitor the dynamics of BCR-regulated ubiquitylation. To enable accurate quantitation of BCR-regulated signaling, we employed the approach of stable isotope labeling by amino acids in cell culture (SILAC) (Ong *et al*, 2002). We used A20 cells, a murine B-cell lymphoma cell line that expresses surface immunoglobulin receptors of the IgG2a isotype, as a model system for investigating BCR signaling (Singh *et al*, 2005). Cells were isotopically labeled using “light” (Lys0, Arg0), “medium” (Lys4, Arg6), or “heavy” (Lys8, Arg10) amino acids, and signaling was analyzed 5 and 15 min after BCR stimulation. The signaling was activated by cross-linking BCRs with the F(ab′)2 fragment of rabbit anti-mouse IgG (α-IgG) for analyzing phosphorylation and ubiquitylation signaling (Fig1A), or biotin-labeled α-IgG F(ab′)2 for investigating the dynamics of BCR signalosomes (Fig1B). Activation of BCR signaling was verified by monitoring phosphorylation of key downstream kinases—BTK1, ERK1/2, and AKT1—using antibodies that recognize activation-state-specific phosphorylation sites on these proteins (Fig1C). All proteomic experiments were performed using liquid chromatography coupled to tandem mass spectrometry (LC-MS/MS). Samples were analyzed on a high-resolution quadrupole Orbitrap (Q-Exactive) mass spectrometer, and the raw data were processed with MaxQuant (Cox & Mann, 2008). At least two biological replicates were performed for each set of experiments.

Analysis of BCR-induced phosphorylation and ubiquitylation. A20 cells were isotopically labeled using the SILAC approach. Control “light” labeled cells were mock-treated, and “medium” and “heavy” labeled cells were stimulated with α-IgG F(ab′)2 for 5 and 15 min, respectively. Di-Gly-modified (ubiquitylated), tyrosine-phosphorylated peptides were enriched sequentially using di-Gly-lysine- and phosphotyrosine-specific antibodies. Phosphorylated peptides were separately enriched using TiO_2_-based chromatography. All samples were analyzed using high-resolution mass spectrometry.

Strategy for analyzing the dynamics of BCR signalosomes. “Medium” and “heavy” SILAC-labeled A20 cells were stimulated with biotinylated α-IgG F(ab′)2 for 5 and 15 min, respectively. Control cells (labeled with “light” SILAC) were mock-treated. Proteins associated with biotinylated α-IgG F(ab′)2-bound BCR signalosomes were affinity-enriched using streptavidin, separated by SDS–PAGE, and analyzed by LC-MS/MS.

Validation of BCR signaling activation. Stimulation of BCR signaling in A20 cells was confirmed using the indicated phosphorylation site-specific antibodies that recognize the activated forms of BTK, ERK1/2, and AKT kinases.

### Analysis of BCR signalosome dynamics

**Figure 2:**
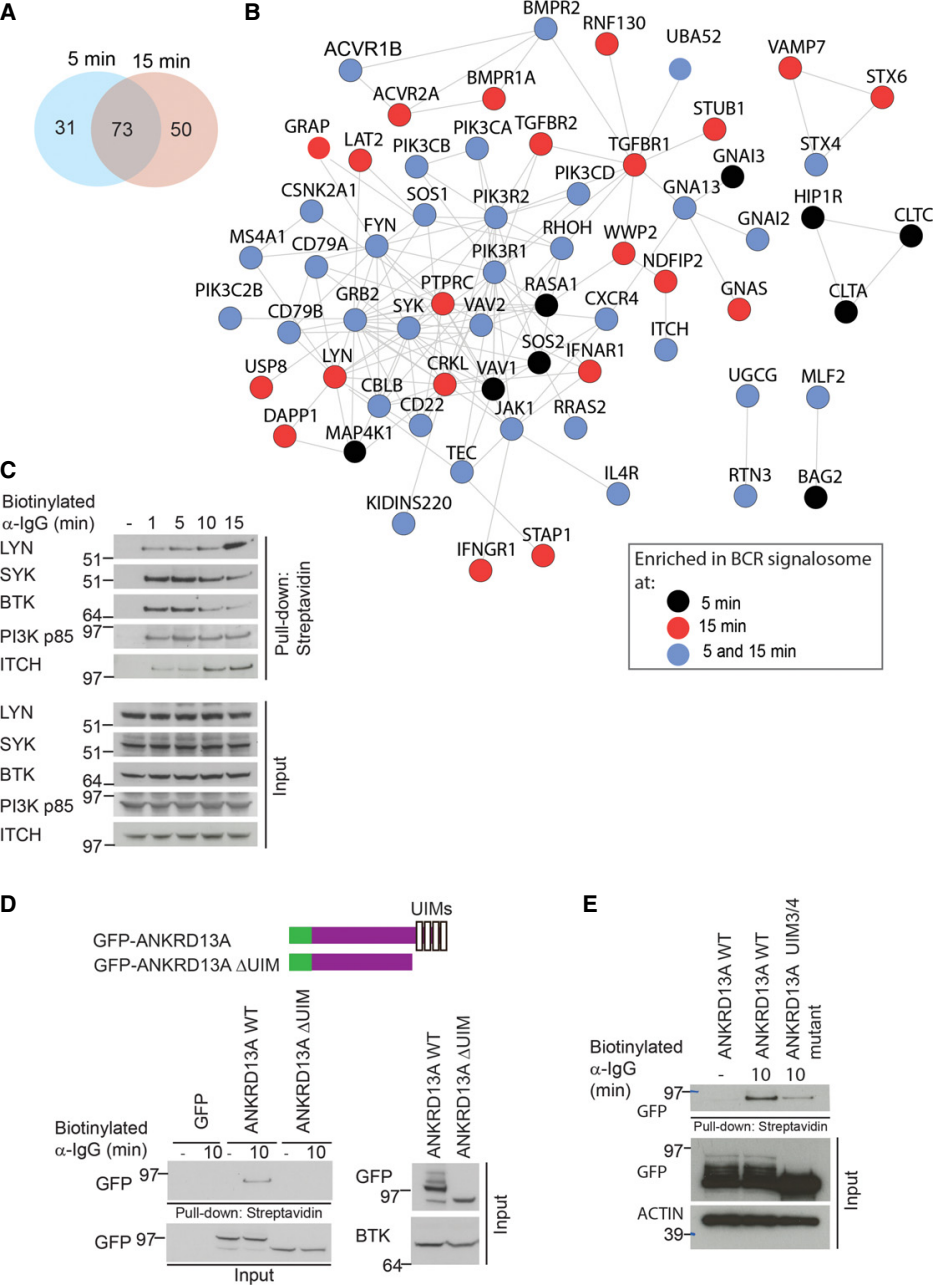
Proteomic analysis of BCR signalosomes

To identify proteins that are present in BCR signalosomes, we cross-linked BCRs with biotin-labeled α-IgG F(ab′)2 and the associated protein complexes (signalosomes) were affinity-purified using streptavidin-conjugated agarose beads (Fig1B). In parallel, control pull-downs were performed by incubating the beads with lysates from unstimulated A20 cells to identify proteins that nonspecifically associated with the beads. The relative quantitation of proteins by SILAC approach allowed us to distinguish proteins that were enriched in BCR signalosomes from the nonspecific binders that were also detected in the control samples. The relative peptide intensities of BCR components indicated that a similar amount of BCRs were present in the signalosomes isolated at 5 and 15 min (Supplementary Fig S1). For determining BCR-specific interactors, we required that: (i) interacting protein should be present in minimum two interactome experiments with a SILAC ratio ≥ 2 in at least one of the time point, and (ii) the median SILAC ratio of interacting proteins should be more than the median + 2SD of the background binding proteins. From four replicate experiments, we identified over 3,000 proteins, of which 154 were specifically enriched in BCR signalosomes (Supplementary Table S1). Out of 154 proteins, 73 proteins were enriched at both the time points, whereas 31 and 50 proteins were enriched at 5 and 15 min of BCR stimulation, respectively (Fig2A). We observed a good overlap of the signalosome-specific proteins in different experiments, and nearly 80% the proteins were consistently enriched (SILAC ratio ≥ 2) in at least three experiments (Supplementary Fig S2A).

A The Venn diagram shows the overlap between the proteins that were enriched in BCR signalosomes at 5 and 15 min.

B A network view of proteins present in BCR signalosome. Proteins are color-coded based on their association with BCR signalosomes at 5 or 15 min after BCR cross-linking.

C Validation of the dynamic association of proteins with BCR signalosomes. A20 cells were stimulated with biotinylated α-IgG F(ab′)2 for the indicated time points, the signalosomes were isolated by streptavidin pull-downs, and enrichment of the indicated proteins was analyzed by immunoblotting.

D, E ANKRD13A associates with BCR signalosomes. A20 cells were transiently transfected with GFP-tagged ANKRD13A WT and the ANRKD13A ΔUIM mutant (D), or ANKRD13A WT and ANKRD13A UIM3/4 mutant (E). BCR signalosomes were isolated as described in (C), and the enrichment of ANKRD13A was probed using α-GFP antibody. The expression of GFP-tagged ANKRD13A WT, ANRKD13A ΔUIM, and ANKRD13A UIM3/4 mutants in the input material was verified.

Among the BCR signalosome components were tyrosine kinases, such as SYK, LYN, and FYN, as well as proteins that function as adaptors in BCR signaling, such as GRB2, DAPP1 (BAM32), and the p85 subunit of PI3K (PIK3R2). Analyzing the signalosomes at two different time points allowed us to investigate the dynamics of some of the associated proteins. For example, SOS1 and SOS2 strongly associated with the signalosomes at 5-min time point, whereas USP8, LYN, and DAPP1 interacted more strongly after 15 min of BCR stimulation (Fig2B, Supplementary Table S1). The interaction of several proteins was independently verified by purification of the signalosomes using the above-described approach followed by immunoblotting with SYK, BTK, LYN, p85 of PI3K, and ITCH antibodies (Fig2C). Together, these results demonstrated the validity of our approach for identifying the components of BCR signalosomes.

Notably, many of the identified signalosome components were either not implicated in BCR signaling, or it was not known that they are constituents of BCR signalosomes. For example, the Kelch domain-containing proteins KLHDC2 and KLHL6 were present in BCR signalosomes (Supplementary Table S1). KLHL6 is mutated in chronic lymphocytic leukemia (CLL) patients (Puente *et al*, 2011), it is an important regulator of B-cell development, and BCR signaling is impaired in KLHL6-deficient cells (Kroll *et al*, 2005). The rapid association of KLHL6 and KLHDC2 with BCR signalosomes indicates that they may participate in the early steps of the receptor signaling. Sprouty-related, EVH1 domain-containing protein 1 (SPRED1) was identified as a dynamic component of BCR signalosomes. Strikingly, SPRED1 associated with the signalosomes more strongly after 15 min compared to 5 min of BCR stimulation. SPRED1 is a negative regulator of the RAS–MAPK signaling pathway downstream of receptor tyrosine kinase signaling (Wakioka *et al*, 2001), and germline loss-of-function mutations in SPRED1 cause neurofibromatosis 1-like syndrome in humans (Brems *et al*, 2007). However, to our knowledge, SPRED1 has not been implicated in BCR signaling previously. Combined deletion of SPRED1 and 2 in mice results in embryonic lethality at embryonic days 12.5 to 15.5 with marked subcutaneous hemorrhage, edema, and dilated lymphatic vessels filled with erythrocytes (Taniguchi *et al*, 2007). Intriguingly, this phenotype resembles the phenotypes of the knockouts of SYK and SLP-76, which play important roles in BCR signaling. Our results suggest that SPRED1 might regulate the RAS–MAPK signaling axis downstream of BCR activation and might play a role in immune signaling.

To further illustrate the usefulness of our approach for identifying previously uncharacterized components of BCR signalosomes, we validated the association of ANKRD13A using complementary techniques (Fig2D and E). The ANKRD13 family proteins (consisting of ANKRD13A, B, and C) were recently identified as epidermal growth factor receptor (EGFR)-interacting proteins that regulate the endocytosis of EGFR (Tanno *et al*, 2012). The function of ANKRD13 in EGFR endocytosis depends on their ubiquitin-interacting motifs (UIMs) (Tanno *et al*, 2012). To confirm the interaction between BCR signalosomes and ANKRD13A, we exogenously expressed GFP-tagged ANKRD13A (ANKRD13A WT) and a mutant form of the protein lacking all four UIMs (ANKRD13A ΔUIM) in A20 cells. ANKRD13A WT associated with the signalosomes isolated from BCR-stimulated cells (Fig2D), and the deletion of the UIMs abrogated this interaction, indicating that ANKRD13A WT associates with BCR signalosomes via its C-termini containing 4 UIMs. To further investigate whether this was through its binding to ubiquitylated components of BCR signalosome, we generated ANKRD13A mutant in which point mutations were introduced in UIM3/4, which have previously been shown to abolish the interaction of these UIMs with ubiquitin (Tanno *et al*, 2012). ANKRD13A UIM3/4 mutant showed a reduced binding to BCR signalosome compared to ANKRD13A WT (Fig2E). These results are consistent with the previously published study (Tanno *et al*, 2012) and suggest a role of UIM3/4 in the binding of ANKRD13A to ubiquitylated proteins; however, further work is required to delineate the detailed mechanisms of ANKRD13A recruitment to BCR signalosomes.

### BCR-regulated phosphoproteome

**Figure 3:**
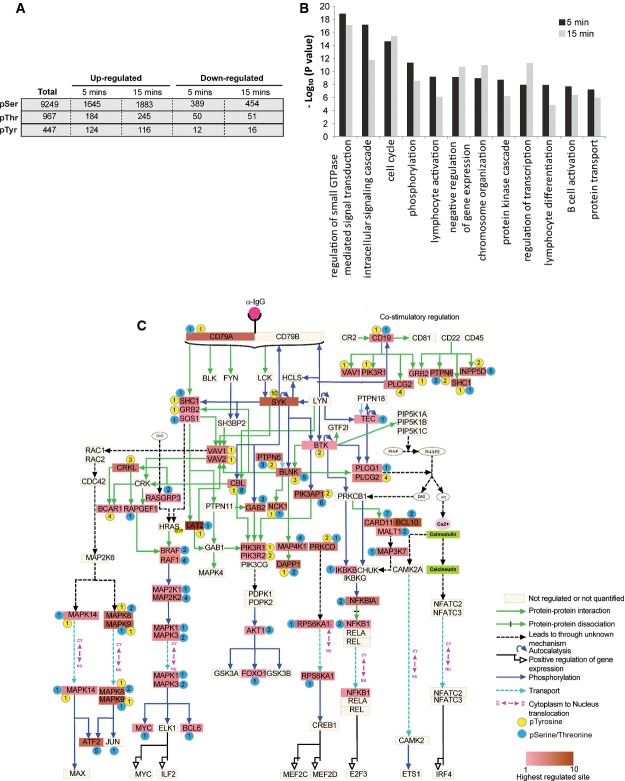
Analysis of BCR-regulated phosphorylation

To obtain further insights into BCR signaling, we monitored the dynamics of protein phosphorylation after BCR stimulation. We quantified 10,663 phosphorylation sites with high confidence (localization probability of ≥ 0.75, Andromeda score ≥ 40, PEP score ≤ 0.01), of which 4.1% were Tyr phosphorylation sites and the remaining were Ser or Thr phosphorylation sites (Supplementary Table S2). Phosphorylation of 1,829 Ser/Thr sites and 124 Tyr sites was increased (≥ 2-fold) after 5 min of BCR stimulation (Fig3A), and the changes observed in two independent replicate experiments correlated significantly (*R* = 0.86 for both 5 and 15 min) (Supplementary Fig S2B). The magnitude of increase in Tyr phosphorylation was significantly greater after 5 min of the stimulation (Wilcoxon test, *P* = 7.63e-15) than the increase in Ser/Thr phosphorylation (Supplementary Fig S3A). While Tyr phosphorylation peaked (average increase 4.0-fold) after 5 min and started to decline (mean increase 3.3-fold) within 15 min of the receptor activation, phosphorylation of Ser/Thr sites was sustained for longer periods (mean increase ∽1.6-fold at both the time points) (Supplementary Fig S3B). Among the proteins with BCR-induced phosphorylation sites were more than 80 protein kinases and 25 ubiquitin ligases (Supplementary Table S3), many of which have not been implicated in BCR signaling. Gene Ontology biological processes (GOBP) term enrichment analysis showed the enrichment of the terms related to receptor signaling and B-cell biology, such as “regulation of small GTPase-mediated signal transduction,” “intracellular signaling cascade,” “lymphocyte activation,” “lymphocyte differentiation,” and “B-cell activation,” among others (Fig3B, and Supplementary Table S4).

An overview of the BCR-regulated phosphoproteome. The table shows the number of the identified phosphorylation sites, and the number of sites that were up- (≥ SILAC ratio 2) or downregulated (≤ SILAC ratio 0.5) after 5 and 15 min of BCR stimulation.

Functional annotation of the BCR-upregulated phosphoproteome. The bar graph shows GO biological processes (GOBP) terms that were significantly enriched in BCR-upregulated phosphoproteome.

A diagram of the BCR signaling pathway. The proteins with upregulated phosphorylation sites in the pathway are colored, and the color gradient indicates the magnitude of phosphorylation increase. The modified amino acids (tyrosine or serine/threonine) are color-coded as circles, and the numbers of BCR-upregulated sites are indicated within the circles. Proteins with light yellow color were either not quantified or did not contain BCR-upregulated phosphorylation.

A network analysis of the BCR-upregulated tyrosine phosphoproteome revealed that most of the regulated proteins were involved in the early steps of BCR signaling (Supplementary Fig S3C). Among the proteins with rapidly upregulated tyrosine phosphorylation sites were tyrosine kinases and signaling adaptors, such as SYK, BTK, LAT2, SHC1, LNK (SH2B3), SLP65 (BLNK), DAPP1, and SCIMP. In addition, we observed increased tyrosine phosphorylation of several other proteins whose roles in BCR signaling are not known, including E3 ubiquitin ligase HECTD1; deubiquitylases USP15 and OTUD4; and ZC3HAV1, which is a potential regulator of NF-κB signaling (Hayakawa *et al*, 2011).

The class IIa histone deacetylases (HDAC4, 5, 7, and 9) bind to the myocyte-specific enhancer factor 2 (MEF2) family of transcription factors to repress the expression of their target genes. Notably, increased phosphorylation was observed on all four members of the MEF2 family (MEF2A, B, C, and D), as well as on HDAC5 and 9 (Supplementary Table S2). MEF2C is required for B-cell proliferation and survival after antigen receptor stimulation (Wilker *et al*, 2008), and MEF2B is frequently mutated in diffuse large B-cell lymphoma (DLBCL) and follicular lymphoma (Mahrour *et al*, 2008; Morin *et al*, 2011; Ying *et al*, 2013), but the extent to which MEF2 family transcription factors are modulated by BCR-induced PTMs was not fully known. Our results suggest that by modulating the phosphorylation of the MEF2 proteins, and the associated HDACs, BCR signaling may regulate their function in gene transcription.

BCR signaling involves activation of three key signaling pathways: the NF-κB pathway, the RAS–MAPK pathway, and the PI3K–AKT pathway. The members of these signaling pathways were prominently represented in our datasets as indicated by their increased phosphorylation (Fig3C). Notably, increased phosphorylation was found on all three subunits of the CBM (CARD11-BCL10–MALT1) complex, which is a critical regulator of antigen receptor-induced NF-κB activation. Antigen receptor-stimulated phosphorylation of BCL10 on S138 negatively impacts the activation of NF-κB signaling (Wegener *et al*, 2006; Zeng *et al*, 2007). We observed a weak increase (1.3- and 1.6-fold at 5 and 15 min, respectively) in phosphorylation of this site. Interestingly, phosphorylation of BCL10 S171 and of S186/S189 increased much more robustly (S171 10-fold and S186/189 27-fold) (Supplementary Table S2), suggesting that these phosphorylation sites may have distinct regulatory functions than does S138 phosphorylation. Receptor activation-induced assembly of the CBM complex activates the IKK complex, which then activates NF-κB signaling by phosphorylating and degrading its inhibitor IκB. Consistent with this model, we observed a dramatic increase in phosphorylation of IκB-α (at S32 and S36) at 5 min followed by a rapid decrease within 15 min (Supplementary Table S2). Recently, IκB-δ (also known as IκBNS) has been identified as a regulator of BCR signaling (Touma *et al*, 2011). Interestingly, we note that IκB-α and IκB-δ show different phosphorylation dynamics. In contrast to a rapid and transient phosphorylation of IκB-α (19.5-fold at 5 min and 2-fold at 15 min), phosphorylation of IκB-δ on S30 and S35 was increased less markedly, but was more sustained. The differential phosphorylation dynamics of these proteins is in line with previous genetic data suggesting that IκB-α and IκB-δ have unique, nonredundant functions in BCR signaling (Touma *et al*, 2011).

The functional roles of rapid, stimulus-dependent increase in phosphorylation are well known in cell surface receptor signaling. However, the functional significance of stimulus-regulated dephosphorylation is less well studied. Among the few proteins that showed decreased tyrosine phosphorylation were adaptor proteins BTLA, NCKIPSD, and PAG1. Tyrosine-phosphorylated PAG1 binds to the tyrosine kinase Csk, which is a major negative regulator of the Src family kinases, and it is involved in the negative regulation of TCR signaling (Brdicka *et al*, 2000). Consistent with a prominent role of Src family kinases in BCR signaling, phosphorylation of PAG1 on Y386 was reduced more than 10-fold within 15 min of BCR activation. Interestingly, we also found many proteins in which BCR stimulation resulted in both increased and decreased phosphorylation of distinct sites on the same protein (Supplementary Table S2). These included proteins with diverse functions, such as the transcription regulators FOXO3, CIC, MEF2C, and JUND; adaptor proteins SCIMP and BTLA; E3 ubiquitin ligases HECTD1 and HUWE1; and protein kinases PAK2, MARK2, ULK1, and PRKD2, among others. Identification of a large number of proteins containing oppositely regulated phosphorylation sites indicates that concurrent phosphorylation and dephosphorylation may be a frequently used mechanism for switching protein functions.

### RAB7A phosphorylation affects subcellular localization and effector binding

**Figure 4:**
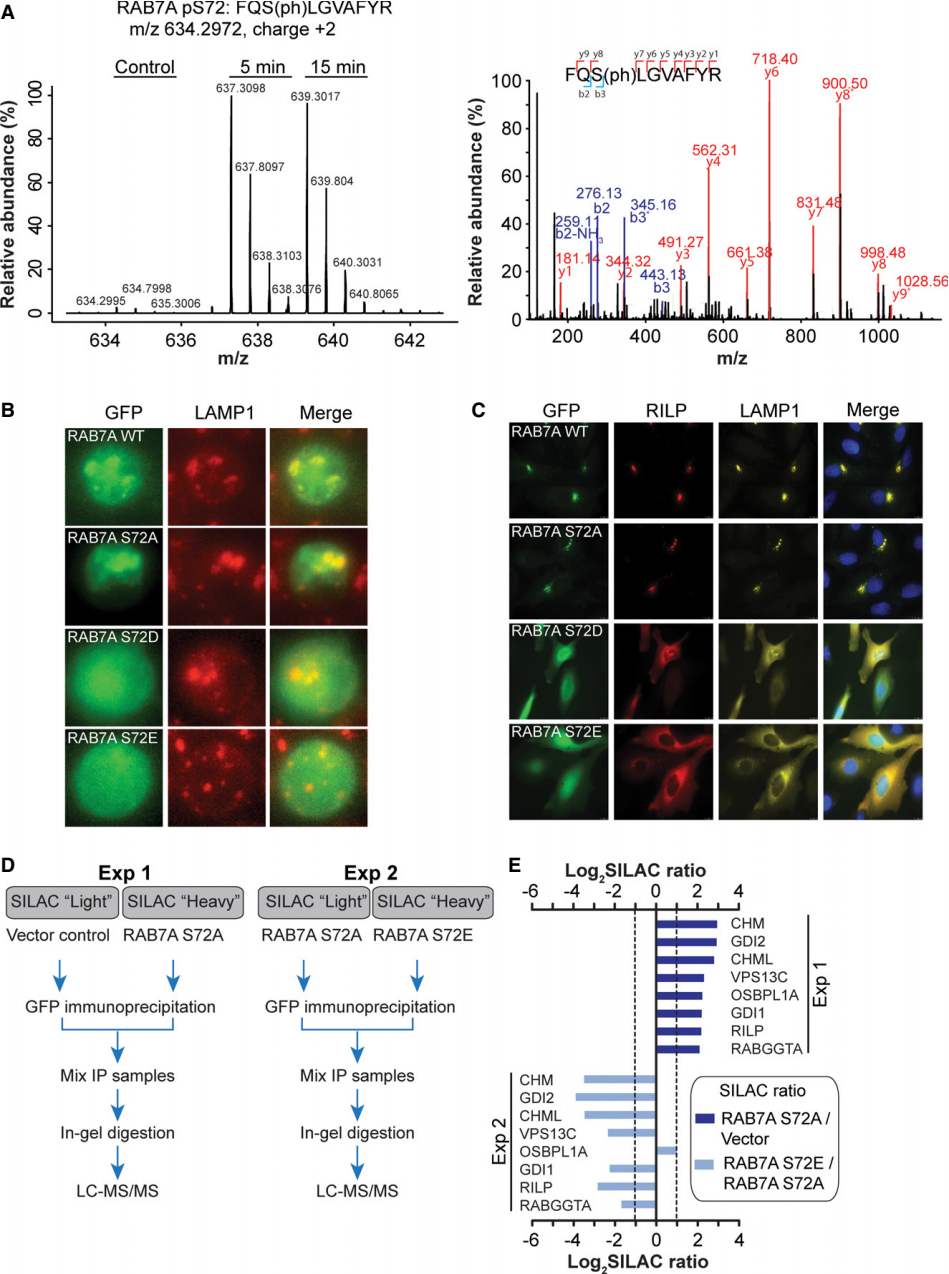
BCR-induced phosphorylation of RAB7A regulates its subcellular localization and effector binding

RAB7A is an important regulator of early-to-late maturation of endosomes and endo-lysosomal trafficking (Zhang *et al*, 2009), and it cycles between an endomembrane-bound form and a nonendomembrane-bound form (Cantalupo *et al*, 2001). We found a dramatically increased (> 20-fold) phosphorylation of RAB7A on S72 in BCR-stimulated cells (Fig4A). To assess the function of this phosphorylation site, we generated phosphomimetic (S72D and S72E) and nonphosphorylatable (S72A) mutants of RAB7A and analyzed their subcellular localization in primary B cells and in HeLa cells. In primary B cells, the RAB7A S72A mutant and wild-type RAB7A localized to endo-lysosomal compartments as indicated by their co-localization with lysosome-associated membrane glycoprotein 1 (LAMP1), a marker for late endosomes/lysosomes (Fig4B). In contrast, the phosphomimetic mutants of RAB7 were diffusely localized. As observed previously (Bucci *et al*, 2000; Cantalupo *et al*, 2001), the overexpression of RAB7A or RAB7A S72A mutant together with its effector RILP (rab-interacting lysosomal protein) in HeLa cells resulted in the formation of enlarged lysosomal structures (Fig4C, and Supplementary Fig S4). In contrast, overexpression of RAB7A S72E and RAB7A S72D with RILP in HeLa cells showed a loss of their endo-lysosomal localization; instead, the mutant proteins were diffusely localized in the cytoplasm. These results suggest that phosphorylated RAB7A is unable to localize to endo-lysosomal compartments.

Identification of BCR-inducible RAB7A S72 phosphorylation. The MS spectrum (the left panel) shows the relative abundance of RAB7A S72 containing phosphopeptide at 5 and 15 min after the stimulation, and the MS/MS spectrum (the right panel) shows fragment ions of the identified peptide.

Subcellular localization of RAB7A phosphorylation mutants in B cells. Primary B cells were isolated from mouse spleen and infected with retroviruses expressing RAB7A WT or the indicated mutants fused with GFP. Cells were fixed with paraformaldehyde and stained with α-LAMP1, and epifluorescence images were acquired. Images show maximum projection of *z*-stacks of the cells.

Subcellular localization of RAB7A phosphorylation mutants in HeLa cells. The cells were co-transfected with FLAG-RILP and RAB7A or the indicated mutants fused with GFP. Cells were fixed, permeabilized, and stained with α-FLAG and α-LAMP1 antibodies to detect the exogenously expressed RILP and the endogenous LAMP1.

Strategy for identifying RAB7A interacting proteins. In experiment 1 (Exp 1), SILAC-labeled HeLa cells were transfected with GFP-RAB7A- or GFP-expressing plasmid vector to identify RAB7A interacting proteins. In a separate experiment (Exp 2), the interaction profile of GFP-RAB7A S72E was compared to GFP-RAB7A S72A.

Interaction profile of the RAB7A and the mutants. The bar graph shows the logarithmized SILAC ratios of the indicated RAB7A-binding proteins that were enriched (≥ 2-fold) with GFP-RAB7A compared to their levels in control immunoprecipitation (Exp 1), and their relative de-enrichment in GFP-RAB7A S72E compared to GFP-RAB7A S72A (Exp 2).

The co-crystal structure of RAB7A and its effector CHM (also known as REP1) shows that S72 is present within a critical regulatory region and forms a direct contact with CHM (Rak *et al*, 2004). To test the effects of RAB7A phosphorylation on its association with CHM and other endo-lysosomal proteins, we used a SILAC-based interaction screen (Fig4D). While RAB7A S72A strongly associated with the known RAB7A interactors such as CHM, CHML, RILP, and VSP13C, the RAB7A S72E mutant exhibited a markedly reduced interaction with these proteins (Fig4E, and Supplementary Table S5). These results are consistent with our observation that RAB7A S72E and RAB7A S72D fail to localize to endo-lysosomal structures. Together, these results identified a novel role of RAB7A S72 phosphorylation, and the dynamic regulation of this site after BCR activation suggests that it may have a functional role in antigen receptor trafficking or signaling.

### The BCR-regulated ubiquitylome

**Figure 5:**
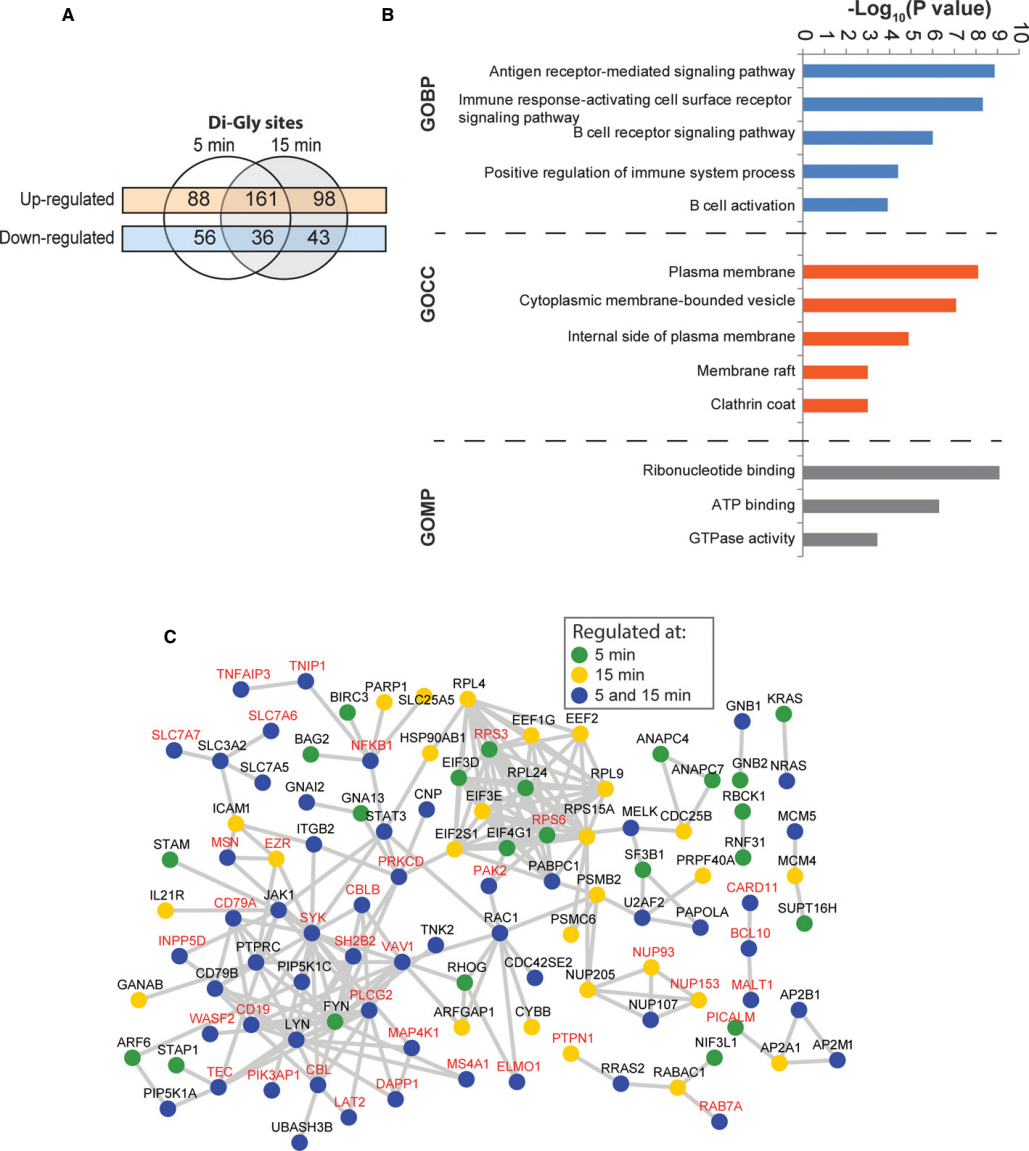
The BCR-induced ubiquitylome

Ubiquitylation serves multiple functions in cell surface receptor trafficking and signaling (Chen & Sun, 2009; Polo, 2012), and we observed that many ubiquitylation-regulating enzymes were associated with BCR signalosomes or contained BCR-induced phosphorylation (Supplementary Tables S1 and S2). To systematically investigate the regulatory scope of ubiquitylation in BCR signaling, we used the di-Gly capture method to enrich ubiquitylated peptides (Wagner *et al*, 2011). We quantified 6,059 ubiquitylation sites after BCR stimulation (Supplementary Table S6). Ubiquitylation of 249 and 259 sites showed an increase (≥ 2-fold) at 5 and 15 min, respectively. A majority of these sites (161) were upregulated at both the time points (Fig5A), with significant correlation between the replicate experiments (Supplementary Fig S2C). GO biological processes (GOBP) term enrichment analysis showed that the ubiquitylated target proteins were involved in diverse functions, such as “antigen receptor-mediated signaling pathway,” “B-cell receptor signaling pathway,” and “B-cell activation” (Fig5B, and Supplementary Table S7), which is consistent with the known function of BCR-regulated ubiquitylation in these processes.

The overlap between BCR-regulated di-Gly-modified (ubiquitylation) sites. The Venn diagram shows the overlap of up- and downregulated sites after 5 or 15 min of BCR stimulation.

The functional annotation of the BCR-upregulated ubiquitylome. The bar graph indicates GO biological processes (GOBP), GO cellular compartments (GOCC), and GO molecular processes (GOMP) terms that were significantly enriched among the proteins containing BCR-upregulated di-Gly-modified sites.

Network analysis of the BCR-regulated ubiquitylome. The network shows proteins that contained BCR-upregulated di-Gly-modified sites at one or both of the time points. The proteins that also harbored BCR-induced phosphorylation are indicated in red type.

Notably, ubiquitylation increased rapidly within 5 min of BCR stimulation and the magnitude of increased ubiquitylation on the several members of BCR signalosome, including the receptor subunits (CD79A, CD79B), downstream kinases (i.e. SYK, LYN), and other signaling components such as LAT2, CBLB, and VAV1, was similar to phosphorylation (Supplementary Table S8). At the later time point, increased ubiquitylation was also observed on tumor necrosis factor alpha-induced protein 3 (TNFAIP3, also known as A20) and TNFAIP3-interacting protein 1 (TNIP1). TNFAIP3 is an important negative regulator of NF-κB signaling in TCR signaling, where it deubiquitylates MALT1 and inactivates the CBM complex to prevent sustained activation of NF-κB signaling (Duwel *et al*, 2009). Thus, a more prominent regulation of these proteins at the later time point is consistent with their known negative regulatory function. Also, consistent with a role for K63-linked ubiquitylation in receptor endocytosis, trafficking, and signaling (Chen & Sun, 2009; Polo, 2012), we observed BCR-induced ubiquitylation of the K63-specific deubiquitylases AMSH-like protease (STAMBPL1) and BRCC3 (BRCC36), as well as a modest increase (∽20%) in K63 linkage-specific ubiquitin peptide.

Among BCR-regulated proteins that were only found in the di-Gly dataset were NRAS, KRAS, and RRAS2 (Supplementary Table S6). NRAS and KRAS were modified on the corresponding lysines K128, and RRAS2 was modified on K106. Ubiquitylation has been implicated in the regulation of RAS signaling (Jura *et al*, 2006; Sasaki *et al*, 2011), and our results show that multiple RAS isoforms are ubiquitylated in response to BCR activation. Increased ubiquitylation was also observed on several sites on the transcription factor STAT3, which plays an important role in B-cell development (Wang *et al*, 2007), as well as on GNAI2, which is also required for B-cell development (Dalwadi *et al*, 2003), and mutated in Burkitt lymphoma and DLBCL (Morin *et al*, 2013). These examples illustrate the effectiveness of our approach in pinpointing BCR-responsive ubiquitylation sites, some of which likely have important regulatory functions.

### Co-occurrence of BCR-induced ubiquitylation and phosphorylation

Notably, many of the ubiquitylated proteins were also co-regulated by phosphorylation (Fig5C and Supplementary Table S9), and several of these were implicated in the receptor-proximal signaling. BCR-induced activation of NF-κB signaling critically impinges on the activation of phospholipase Cγ2 (PLCγ2) and protein kinase C (PKC), which subsequently activate the CBM complex (Thome *et al*, 2010). We found that both phosphorylation and ubiquitylation were rapidly induced on PLCγ2 and PKC, as well as on all three components of the CBM complex—CARD11, BCL10, and MALT1. While these proteins were known to have important functions in BCR signaling, most of the regulated sites identified here were not known to be regulated by BCR stimulation.

Increased phosphorylation of NF-κB1 is a hallmark of the activation of the canonical NF-κB pathway. Interestingly, we found BCR-induced ubiquitylation of NF-κB1 at K852, which is located within the DEATH domain of NF-κB1. Ubiquitylation of this site peaked at 5 min and started to decline after 15 min of the receptor activation. Upon TCR activation, ubiquitin ligase PELI1 negatively regulates c-REL through K48-linked ubiquitylation, and genetic deficiency in PELI1 causes hyperactivation of T cells and development of autoimmunity (Chang *et al*, 2011). We found BCR-induced phosphorylation and ubiquitylation on PELI1 (Supplementary Table S9). Consistent with a negative regulatory role of PELI1 in TCR signaling, ubiquitylation and phosphorylation of PELI1 was more robustly increased at the later time points of BCR activation, implying that it may have a similar regulatory function in BCR signaling. In addition to the regulators of the NF-κB pathway, ubiquitin ligases CBLB and CBL contained BCR-induced phosphorylation and ubiquitylation sites. Notably, phosphorylation and ubiquitylation of CBLB was more pronounced than that of CBL. A large number of kinases involved in the MAPK signaling pathway showed increased phosphorylation, but only MAP4K1 (also known as MEKKK1 or HPK1) showed both increased phosphorylation and ubiquitylation. Together, these results provided a first glimpse of co-regulated phosphorylation and ubiquitylation in BCR signaling.

### BCR stimulation induces linear ubiquitylation of BCL10

**Figure 6:**
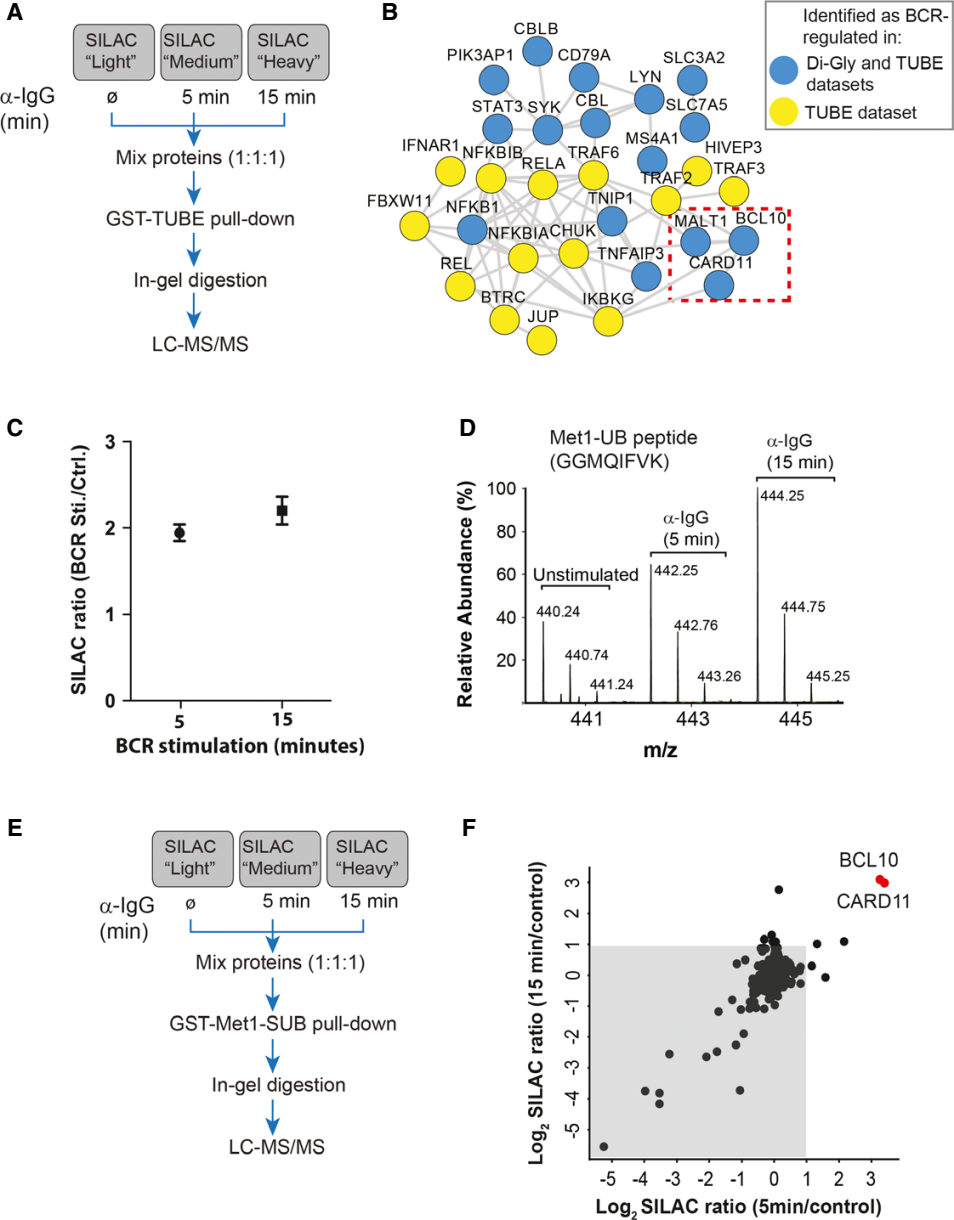
BCR stimulation induces linear ubiquitylation of BCL10

To complement the results obtained with the di-Gly profiling approach, we used recombinant tandem ubiquitin-binding entities (TUBEs) (Hjerpe *et al*, 2009) and SILAC-based proteomics to identify proteins that were ubiquitylated in response to BCR activation (Fig6A). The enriched proteins were separated on SDS–PAGE, proteolyzed, and analyzed by MS. In total, we identified 2,253 proteins in the TUBE pull-downs, of which 66 showed ≥ 2-fold increased enrichment in BCR-stimulated samples (Supplementary Fig S5 and Supplementary Table S10). Many of the BCR-regulated ubiquitylated proteins identified in these experiments were also identified as BCR-dependent ubiquitylated in our di-Gly dataset (Fig6B, and Supplementary Fig S5), including all three components of the CBM complex. A few proteins, such TRAF6, TRAF2, and ITPR3, were reproducibly identified as BCR-regulated (SILAC ratio ≥ 2-fold) only in the TUBE-based pull-downs. Because TUBEs bind to proteins containing diverse types of ubiquitin linkages, we queried our data for the presence of peptides derived from specific ubiquitin linkages. Notably, the relative abundance of linear ubiquitin (Met1-UB) peptide was increased in BCR-stimulated cells (Fig6C and D). The relative abundance of linear ubiquitin peptide was more prominently increased in SDS–PAGE gel fractions that contained higher molecular weight proteins (Supplementary Fig S6A), suggesting that it likely resulted from linearly ubiquitylated proteins rather than from unconjugated linear ubiquitin chains.

A Strategy for the identification of BCR-regulated ubiquitylated proteins. SILAC-labeled A20 cells were stimulated with α-IgG F(ab′)2 for the indicated times, and ubiquitylated proteins were affinity-enriched using tandem ubiquitin-binding entities (TUBE)-based pull-downs

B Interaction network of BCR-induced ubiquitylated proteins. The network shows interaction among the proteins that were significantly enriched in TUBE pull-downs from BCR-stimulated cells compared to proteins pulled down from mock-treated control cells. Blue circles indicate the proteins which were also identified as BCR-upregulated in our di-Gly dataset. The dotted box indicates members of the CBM complex.

C BCR stimulation increases the abundance of Met1-linked ubiquitin (Met1-UB). The plot shows the SILAC ratios of peptide corresponding to Met1-UB chains in the TUBE pull-downs from (A). The error bars represent mean ± SEM of the SILAC ratios.

D BCR stimulation increases the abundance of linear ubiquitin peptide. The MS spectrum shows the relative abundance of the peptide (GGMQIFVK) corresponding to Met1-UB in TUBE pull-downs from unstimulated cells, or after 5 or 15 min of BCR stimulation.

E, F Identification of BCR-regulated linear ubiquitylated proteins. Schematic presentation of the strategy used for SILAC-based Met1-SUB pull-downs (E). The scatter plot shows proteins identified in Met1-SUB pull-downs (F). The gray background indicates proteins that were identified in the pull-downs independent of BCR stimulation, and the red dots indicate proteins that were enriched after 5 and 15 min of BCR stimulation.

The BCR stimulation-dependent increase in linear ubiquitin prompted us to identify possible target(s) of linear ubiquitylation in this system. We used quantitative proteomics and the Met1-specific ubiquitin binder (Met1-SUB) (Fiil *et al*, 2013) to identify BCR-induced linearly ubiquitylated protein(s) (Fig6E). Met1-SUB is comprised of the GST-tagged UBAN domain of NEMO, which binds to linear ubiquitin with about 100-fold higher affinity than to K63-linked and other types of ubiquitin chains (Lo *et al*, 2009; Rahighi *et al*, 2009). This strategy identified BCL10 and CARD11 as BCR stimulation-specific interactors in Met1-SUB pull-downs (Fig6F, and Supplementary Table S11). Notably, although BCL10 is a 28-kDa protein, it was detected in much higher molecular weight SDS–PAGE fractions (Supplementary Fig S6B), suggesting that it was likely posttranslationally modified. Also, the peptide corresponding to linear ubiquitin was enriched in the same higher molecular weight gel fractions. Furthermore, when the Met1-SUB pull-down experiments were repeated under more stringent washing conditions (650 mM NaCl), we found BCR activation-dependent enrichment of BCL10, but not of CARD11 (data not shown), suggesting that BCL10 is a major target of BCR-induced linear ubiquitylation.

**Figure 7:**
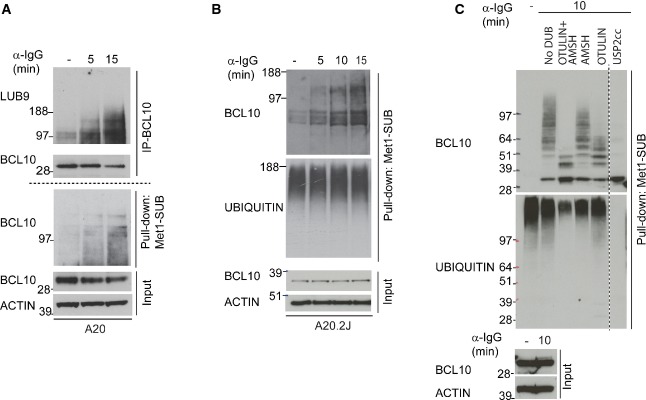
Validation of BCL10 linear ubiquitylation

BCR-induced linear ubiquitylation of BCL10 was independently confirmed using the LUB9 antibody that specifically binds to linear ubiquitin chains (Fig7A) (Sasaki *et al*, 2013). Additionally, using Met1-SUB we affinity-enriched linearly ubiquitylated proteins and observed a robust and selective enrichment of BCL10 from BCR-stimulated A20 and A20.2J cells (Figs7A and B). Linear ubiquitylation of BCL10 was further verified by using linkage-specific DUBs (Mevissen *et al*, 2013). Treatment of Met1-SUB immunoprecipitates with the catalytic core of USP2 (USP2 cc), which functions as unspecific DUB, completely eliminated the higher molecular weight ubiquitylated form of BCL10 (Fig7C). In contrast, treatment of the same pull-down material with OTULIN, a linear ubiquitin-specific DUB, collapsed the higher molecular weight BCL10 to lower molecular weight species; however, it was not able to completely convert the modified BCL10 to the unmodified form. Treatment with AMSH (which cleaves K63-linked ubiquitin chains) and OTULIN in combination led to a more pronounced reduction in the poly-ubiquitylated form of BCL10 (Fig7C), indicating that it was likely modified with both the linear and K63-linked ubiquitin chains.

A, B Validation of BCR stimulation-dependent linear ubiquitylation of BCL10. BCL10 was immunoprecipitated from BCR-stimulated and unstimulated control A20 cells and immunoblotted with LUB9 antibody that binds to linear ubiquitin (A, the upper panel). From the same cell lysates, linear ubiquitylated proteins were isolated using Met1-SUB and probed with BCL10 (A, the lower panel). The latter approach was also used to confirm BCR-induced linear ubiquitylation of BCL10 in A20.2J cells (B). The blots at the bottom of the figure show the expression levels of BCL10 and actin in the input material used for BCL10 immunoprecipitation and Met1-SUB pull-downs.

C BCL10 ubiquitylation is sensitive to linear and K63 linkage-specific deubiquitylases. Linearly ubiquitylated proteins were isolated from BCR stimulated cells with Met1-SUB, treated with the indicated deubiquitylases, and subsequently immunostained with antibodies recognizing BCL10, or ubiquitin. The blots at the bottom of the figures show the expression levels of BCL10 and actin in the input material used for Met1-SUB pull-downs.

### Function of TRAF6 and HOIP in BCL10 ubiquitylation and NF-κB activation

**Figure 8:**
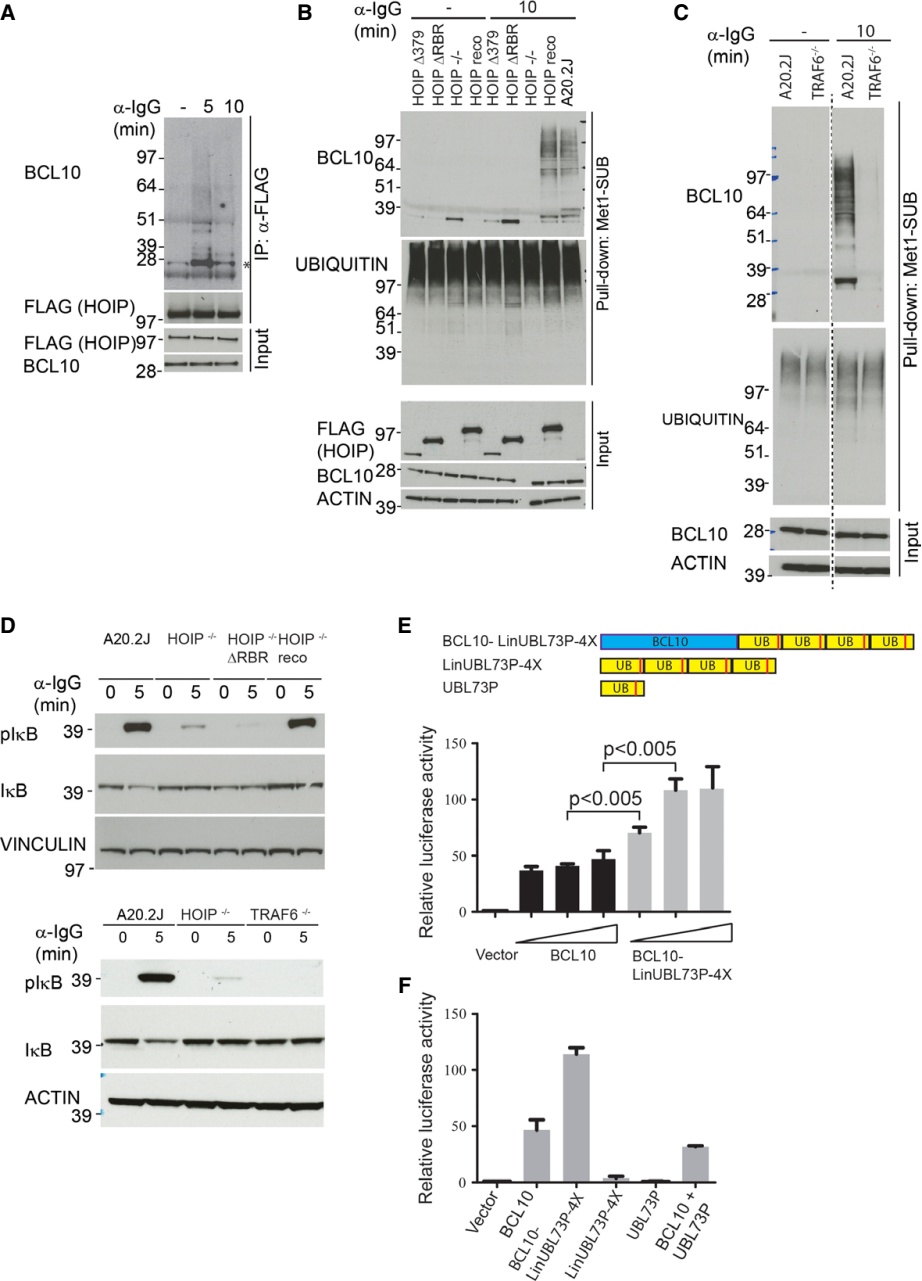
Functional analysis of BCL10 linear ubiquitylation

LUBAC is the only linear ubiquitin ligase known to date (Iwai *et al*, 2014), and HOIP (also known as RNF31) is the core catalytic subunit of this complex. We observed a BCR activation-dependent interaction between BCL10 and HOIP (Fig8A). To investigate whether HOIP is required for linear ubiquitylation of BCL10, we used HOIP-deficient A20.2J cells (HOIP^−/−^) (Hostager *et al*, 2011), HOIP^−/−^ cells reconstituted with HOIP full-length (HOIP reco), or HOIP lacking the RBR (RING1-IBR-RING2) domain (HOIP ΔRBR), or HOIP lacking both the RBR and LDD (linear ubiquitin chain determining domain) domains (HOIP Δ379). It has previously been demonstrated that the RBR and LDD domains are required for the catalytic activity of HOIP (Sasaki *et al*, 2013). Notably, linear ubiquitylation of BCL10 was abolished in HOIP^−/−^ cells, and in cells expressing catalytically inactive HOIP mutants (Fig8B), demonstrating a key role of HOIP in BCR-induced linear ubiquitylation of BCL10.

A HOIP interacts with BCL10. A20.2J HOIP^−/−^ cells reconstituted with the full-length FLAG-HOIP were stimulated with α-IgG for the indicated times, HOIP was immunoprecipitated with α-FLAG antibody, and the immunoprecipitates were immunostained with BCL10 antibody. The amounts of immunoprecipitated HOIP, and equal expression of FLAG-HOIP and BCL10 in whole-cell lysates used for the IPs, are shown in the lower blots. The asterisk indicates unmodified BCL10.

B HOIP is required for BCL10 linear ubiquitylation. A20.2J, A20.2J HOIP^−/−^, and A20.2J HOIP^−/−^ cells expressing the HOIP full-length or the indicated HOIP mutants were stimulated with α-IgG followed by Met1-SUB pull-down and immunoblotting as described in Fig7A.

C TRAF6 is required for BCL10 linear ubiquitylation. A20.2J wild-type and TRAF6^−/−^ cells were stimulated with α-IgG for 10 min, and linear ubiquitylated proteins were pulled down with Met1-SUB and immunoblotted with BCL10 and ubiquitin antibodies. Equal expression of BCL10 and actin was verified in the input material.

D HOIP and TRAF6 function is important for BCR-induced IκB phosphorylation. A20.2J wild-type, HOIP^−/−^, TRAF6^−/−^, and HOIP^−/−^ cells expressing HOIP ΔRBR were stimulated with α-IgG for the indicated time points, and lysates were immunostained with pIκB and IκB antibodies. Actin and vinculin staining serves as loading control.

E, F BCL10 linear ubiquitin fusion protein activates NF-κB. HEK293T cells were co-transfected with increasing amount (0.5, 1 or 2 μg) of BCL10, or BCL10-LinUBL73P-4X construct together with pNF-κB Luc and pRL-TK Renilla. NF-κB transcriptional activity was measured 24 h later using Dual-Glo® Luciferase Assay System (Promega). In (F), NF-κB transcriptional activity was measured in HEK293T cells co-transfected with 1 μg of the indicated plasmids. Error bars indicate mean ± SEM of 3 (E) or 2 (F) independent experiments. Statistical significance was determined by two-tailed Student's *t*-test.

TRAF6, which catalyzes K63-linked ubiquitylation, has been implicated in the ubiquitylation of the CBM complex (Sun *et al*, 2004). The identification of TRAF6 among BCR-induced ubiquitylated proteins (Fig6B) and the presence of linear as well as K63-linked ubiquitin chains on BCL10 (Fig7C) prompted us to test whether TRAF6 is involved in linear ubiquitylation of BCL10. Strikingly, BCR-induced linear ubiquitylation of BCL10 was abolished in TRAF6 knockout cells (Fig8C), suggesting that TRAF6 functions upstream of LUBAC in this pathway.

Because of the essential role of the CBM complex in antigen receptor-mediated activation of IKK signaling, and robust induction of linear ubiquitylation of BCL10 in response to BCR engagement, we hypothesized that linear ubiquitylation may have a functional role in this pathway. To test this, we investigated BCR-induced phosphorylation of IκB, which is a sensitive marker of IKK activation; in HOIP^−/−^ and HOIP^−/−^ cells expressing HOIP ΔRBR. BCR-induced phosphorylation of IκB was markedly diminished in HOIP^−/−^ cells and in HOIP ΔRBR-expressing cells (Fig8D), suggesting an important function of HOIP in BCR-induced IΚΚ activation. A similar reduction in phosphorylation of IκB was observed in TRAF6^−/−^ cells (Fig8D), suggesting that both LUBAC and TRAF6 contribute to BCR-induced IKK activation.

To test whether linear ubiquitylation of BCL10 is functionally relevant for BCR-induced NF-κB activation, we constructed a fusion protein of BCL10 and four linearly linked DUB-resistant ubiquitin (L73P) moieties (Bekes *et al*, 2013) (BCL10-LinUBL73P-4X). Overexpression of BCL10-LinUBL73P-4X in HEK293T cells more potently activated the NF-κB reporter activity compared to BCL10 alone (Fig8E). Surprisingly, in BCL10-LinUBL73P-4X expressing cell lysates, we noted the appearance of BCL10 bands migrating at lower molecular weight which corresponded to BCL10 fusion proteins with one, two, or three ubiquitin (Supplementary Fig S6C), suggesting that under the experimental conditions used here a portion of DUB-resistant (L73P) linear ubiquitin chains were cleaved. To rule out the possibility that cleaved ubiquitin from BCL10-LinUBL73P-4X may have caused the increased NF-κB activity, we overexpressed LinUBL73P-4X or UBL73P constructs individually. However, no appreciable increase in NF-κB reporter activity was observed in cells expressing LinUBL73P-4X or UBL73P (Fig8F). We obtained similar results when BCL10 was fused to another uncleavable version of ubiquitin (Rahighi *et al*, 2009), further supporting that linear ubiquitylation of BCL10 enhances NF-κB activation (Supplementary Fig S6D).

## Discussion

Engagement of many cell surface receptors, such as antigen receptors and receptor tyrosine kinases, with their cognate ligands leads to the assembly of signaling complexes and activation of downstream signaling. While the importance of orderly assembly of signaling complexes and dynamic regulation of phosphorylation and ubiquitylation is widely known, due to technological challenges these events are rarely studied simultaneously. Here we used BCR signaling as a model to demonstrate that multifaceted analysis of signaling pathways by parallel monitoring of the dynamics of the receptor signalosomes, phosphorylation, and ubiquitylation can provide an integrated view of signaling networks.

Analyzing proteins that associate with signalosomes is a useful approach for identifying proteins that control signal initiation, amplitude, and duration; as such proteins typically function at the receptor level. Proteins that contain stimulus-regulated PTMs, but are not directly binding to receptor signalosomes may control other aspects of signaling, such as the regulation of gene transcription. Thus, parallel investigation of receptor signaling complexes and downstream signaling events can provide indications about the involvement of proteins in different aspects of receptor signaling. We demonstrate that affinity purification of ligand-bound receptors provides an elegant approach to investigate the receptor-associated signaling complexes. However, we were unable to isolate nonactivated BCRs, and thus, we could not distinguish between constitutively interacting proteins from stimulus-dependent interactors. For the same reason, it was not feasible to identify proteins that dissociate from BCR after the receptor activation. Nevertheless, this approach identified many well-known components of BCR signalosome and identified several potential novel components, and we anticipate that similar strategies could be used to characterize other receptor signalosomes.

Remarkably, BCR stimulation affected phosphorylation of about one-fifth of all quantified sites and a quarter of all tyrosine phosphorylation sites, showing that BCR activation causes a rapid and extensive reorganization of phosphorylation signaling networks. Our dataset included most previously known BCR signaling components that function immediately downstream of the receptor, such as tyrosine kinases and signaling adaptor proteins. Several of these proteins were also identified in the signalosomes purified from BCR-stimulated cells. Our results also showed that ubiquitylation is dynamically regulated at hundreds of sites within minutes after BCR stimulation. Identification of a large number of diverse ubiquitylation-regulating proteins, including ubiquitin ligases from the RING, HECT, and TRIM families, indicates that ubiquitylation-dependent signaling has a more pervasive role in BCR signaling than previously known.

Dysregulated BCR signaling has been implicated in various B-cell malignancies (Buchner & Muschen, 2014). Strikingly, a large number of proteins with BCR-regulated phosphorylation and ubiquitylation, such as CARD11, BCL10, HOIP, PIK3R1, ARID1A, MEF2B, GNA13, GNAI2, POU2F2, and KLHL6, are directly associated with B-cell malignancies (Staudt, 2010; Morin *et al*, 2011, 2013; Ying *et al*, 2013; Young & Staudt, 2013; Yang *et al*, 2014). While some of these proteins are well-known components of BCR signaling, the functional roles of several others, such as GNA13, GNAI2, and KLHL6, remain to be investigated. Furthermore, most of the identified BCR-regulated PTM sites on these proteins have not been functionally characterized.

While many BCR signaling components, such as CD79A, CD79B, CBL, and SYK, were identified in all three proteomic screens, numerous others were identified in only one or two proteomic datasets. For example, analysis of BCR signalosomes identified WWP2, ITCH, and TRIM21 ubiquitin ligases as components of BCR signaling; however, these proteins were not found to be regulated by BCR in our phosphorylation and ubiquitylation datasets. In contrast, we identified multiple BCR-induced ubiquitylation and phosphorylation sites on the CBM complex members, and on the deubiquitylase A20, but these proteins were not captured in the signalosome interaction dataset. We used both the di-Gly profiling and TUBE methods to analyze BCR-regulated ubiquitylation as each of these approaches have their own pros and cons. For example, while the di-Gly profiling approach allowed quantifying ubiquitylation in a site-specific manner, this approach is unable to identify sites that are present on peptides that are not easily amenable to shotgun proteomics. Also, this approach is not suited to quantify changes in ubiquitylation that occurs through extension of pre-existing substrate-conjugated ubiquitin molecules. The TUBE-based approach overcomes some of these limitations, but it has its own drawbacks. For example, with this method it is not possible to distinguish between ubiquitylated proteins from nonubiquitylated proteins that are co-purified with ubiquitylated proteins. Also, this method is not suited to quantify changes in ubiquitylation at individual sites; thus, the enrichment is determined by the overall ubiquitylation level of proteins. Thus, combining the di-Gly and TUBE-based approaches could provide complementary information for identifying stimulus-triggered ubiquitylation, as exemplified in this work. We envisage that similar multilayered proteomic investigations could be employed to obtain a systems-wide understanding of other receptor signaling systems.

To illustrate the usefulness of our approach in identifying novel components of BCR signaling, we verified one independent finding (involving ANKRD13A, RAB7A, and BCL10) from each aspect of signaling investigated using proteomic approaches. We showed that ANKRD13A is a novel component of BCR signalosomes. ANKRD13A associates with BCR signalosomes through its UIMs, which preferentially interact with K63-linked ubiquitin chains (Tanno *et al*, 2012), suggesting that it may interact with ubiquitylated components of the signalosomes. Using phosphoproteomic analyses, we showed that BCR stimulation increases phosphorylation of RAB7 S72 and that RAB7A phosphorylation mimetic mutants fail to bind to its effector proteins and to endo-lysosomes. The BCR-regulated phosphorylation site is present in the switch region of RAB7, which is important for its interaction with the effector protein REP1 (Rak *et al*, 2004). A rapid and robust upregulation of RAB7A S72 phosphorylation by BCR stimulation and reduced binding of the RAB7A phosphorylation mimetic mutants to its effectors indicates that BCR signaling could modulate RAB7A function.

Linear ubiquitylation has emerged as an important regulator of NF-κB activation in pro-inflammatory receptor signaling (Iwai *et al*, 2014). Here we demonstrate that BCR signaling also involves linear ubiquitylation and identified BCL10 as a key target of BCR-induced linear ubiquitylation. Linear ubiquitylation of BCL10 was rigorously validated using mass spectrometry, linear ubiquitin binding Met1-SUB, linkage-specific DUBs, linear ubiquitin-specific antibody, and knockout cells. Our results suggest that BCL10 is modified by linear ubiquitin chains as well as by other types of ubiquitin chains, likely including K63-linked chains. This is consistent with our genetic data showing that BCR-induced linear ubiquitylation is abolished in HOIP KO cells and phosphorylation of IκB in these cells is reduced markedly. Our biochemical and genetic data further showed that BCL10 ubiquitylation involves both TRAF6 and LUBAC and that TRAF6 functions upstream of LUBAC in this pathway. These findings are consistent with a recent report showing that most linear ubiquitylation seen following signaling via the Toll-like receptors or IL-1 occurs in conjunction with K63-linked chains (Emmerich *et al*, 2013). This suggests that concurrent modification of proteins with linear and K63-linked ubiquitin may be more widely used mechanism for activating NF-κB signaling.

It is also worth mentioning that the CBM complex is also involved in the activation of NF-κB signaling by TCR and several natural killer (NK) cell receptors, such as NK1.1, Ly49D, Ly49H, and NKG2D (Gross *et al*, 2008; Thome *et al*, 2010; Marion *et al*, 2012). It has been shown that BCL10 is modified with K63-linked ubiquitylation after TCR activation, and this induces the binding of NEMO to the modified BCL10 (Wu & Ashwell, 2008). In light of our observation that BCR activation induces linear (and possibly with K63-linked) ubiquitylation of BCL10 and that NEMO binds to linear and, to lesser extent, K63-linked ubiquitylation (Rahighi *et al*, 2009), it would be interesting to investigate whether linear ubiquitylation is also involved in the activation of NF-κB by TCRs.

While our results identified BCL10 as a novel target of linear ubiquitylation and demonstrated an important function of HOIP in BCR-induced linear ubiquitylation of BCL10 and activation of IKK signaling, they also raise further questions: (i) Why do cells require both TRAF6 and HOIP for IKK activation? (ii) What is the exact mechanism for the recruitment of HOIP to the BCR signaling complexes? and (iii) How does BCL10 linear ubiquitylation activates IKK signaling? Further studies are required to provide insights into these questions.

Germline mutations in HOIP are associated with the activated B-cell-like subgroup of DLBCL (ABC DLBCL), and LUBAC is important for the viability of DLBCL cells (Yang *et al*, 2014). This is consistent with our observation that HOIP is required for BCR-induced full activation of IKK in A20.2J and A20 cells, which develops into ABC DLBCL-like lymphoma when injected into syngenic mice (Donnou *et al*, 2012). Intriguingly, in contrast to B-cell lymphoma cell lines, LUBAC activity is mostly dispensable for BCR signaling in primary lymphocytes (Sasaki *et al*, 2013). While the reasons for these differences are not known, it is plausible that the functional importance of LUBAC is different in B-cell lymphoma cells compared to primary B cells. Also, it has been shown that BCRs composed of different immunoglobulin (Ig) chain types can activate distinct signaling events (Martin & Goodnow, 2002; Wakabayashi *et al*, 2002), and it is plausible that the different Ig types expressed in A20 cells and primary B cells may differently engage LUBAC in the downstream signaling.

In summary, this work demonstrates the effectiveness of combined proteomic analyses for detailed characterization of signaling systems. The dataset provides a proteome-wide, integrated view of BCR signaling and substantially broadens the knowledge about the regulatory protein phosphorylation and ubiquitylation in this system. Classification of many novel BCR-regulated proteins and PTM sites in our dataset should facilitate the investigation of their functional roles in BCR signaling, as well as in B-cell lymphomas.

## Materials and Methods

### Cell culture

A20 and A20.2J cells were cultured in RPMI-1640 while HEK293T and HeLa cells were cultured in DMEM media supplemented with 10% fetal bovine serum, l-glutamine, penicillin, and streptomycin. For SILAC labeling, cells were cultured in the media containing either unlabeled light amino acids (l-lysine; l-arginine), stable isotope labeled “medium” amino acids (l-lysine [4,4,5,5-D4], l-arginine – U-^13^C_6_), or “heavy” amino acids (l-lysine- U-^13^C_6_, ^15^N_2_; l-arginine-U-^13^C_6_, ^15^N_4_) (Cambridge Isotope Laboratories), as described previously (Ong *et al*, 2002). SILAC incorporation efficiency was 97–98% for isotopically labeled amino acids. Cells were cultured at 37°C in a humidified incubator containing 5% CO_2_. A20, HEK293T, and HeLa cells were obtained from American Type Culture Collection (ATCC), and the generation of A20.2J, A20 HOIP^−/−^, and A20.2J TRAF6^−/−^ has been described elsewhere (Kim *et al*, 1979; McKean *et al*, 1981; Rowland *et al*, 2007; Hostager *et al*, 2011).

### Cell stimulation

For analyzing BCR-induced phosphorylation and ubiquitylation signaling, A20-, A20.2J-, and A20.2J-derived cell lines were serum-starved for 4 h. BCRs were stimulated with 20 μg/ml of the F(ab′)2 of rabbit anti-mouse IgG (Jackson ImmunoResearch Laboratories). For the isolation of BCR signalosomes, “medium” and “heavy” labeled cells were stimulated with biotinylated F(ab′)2 rabbit anti-mouse IgG (Jackson ImmunoResearch Laboratories), and unstimulated (without anti-mouse IgG) “light” labeled cells were used as control. After stimulation, cold phosphate-buffered saline (PBS) was immediately added to the cell suspension to stop the stimulation and cells were harvested by centrifugation, washed twice with PBS, and were either frozen in liquid nitrogen or directly processed for subsequent experiments.

### Pull-downs and immunoprecipitation for mass spectrometry analysis

For BCR signalosome analysis, A20 cells were lysed in JS buffer (25 mM HEPES pH 7.5, 150 mM NaCl, 5 mM EGTA, 1% glycerol, 1% Triton X-100, 1.5 mM MgCl_2_) supplemented with protease inhibitors (Complete protease inhibitor cocktail tablets, Roche Diagnostics), phosphatase inhibitors, 1 mM DTT, and 5 mM N-ethylmaleimide (Sigma). Cell debris was pelleted by centrifugation. Approximately 5 mg of protein was mixed with 20 μl streptavidin-conjugated agarose beads and incubated for 2 h at 4°C. The beads were washed four times with ice-cold NET buffer (50 mM Tris–HCl pH 7.5, 150 mM NaCl, 5 mM EDTA, 0.1% Triton X-100), and the bound proteins were eluted using NuPAGE sample buffer (Invitrogen). The proteins were separated by SDS–PAGE, the gel was cut into 10 slices, and proteins were digested in-gel with trypsin (Sigma). Peptides were eluted from the gel pieces and desalted on reversed-phase C18 StageTips (Rappsilber *et al*, 2007).

For TUBE and Met1-SUB pull-downs, cells were lysed with modified RIPA buffer (50 mM Tris pH 7.5, 150 mM NaCl, 1 mM EDTA, 1% NP-40, 0.1% sodium deoxycholate) supplemented with protease inhibitors and 10 mM N-ethylmaleimide (Sigma). Five milligrams of proteins from each SILAC conditions was mixed at 1:1:1 ratios and incubated with recombinant GST-tagged TUBE or GST-tagged Met1-SUB and glutathione-conjugated agarose (GE Healthcare) for 4 h at 4°C on a rotation wheel. The beads were washed three times with ice-cold modified RIPA buffer, and proteins were eluted, separated on SDS–PAGE, and digested in-gel as described above. GST-tagged TUBE and GST-tagged Met1-SUB were purified as described before (Hjerpe *et al*, 2009; Fiil *et al*, 2013).

For analyzing RAB7A interactome, SILAC-labeled HeLa cells were transfected with either GFP-tagged RAB7A S72A, or RAB7A S72E, or plasmid vector expressing GFP. Cells were lysed as described previously (McCray *et al*, 2010). Briefly, cells were lysed in RAB7A IP buffer (20 mM HEPES, 10% glycerol, 0.5% Triton X-100, 150 mM NaCl, 2 mM MgCl_2_) supplemented with protease inhibitor (Roche) and phosphatase inhibitors. GFP-trap (ChromoTek) was used to enrich GFP-tagged RAB7A protein followed by three washes with ice-cold RAB7A IP buffer. The enriched proteins from different SILAC samples were mixed, and the eluent was subjected to in-gel digestion and LC-MS/MS analysis.

### Enrichment of phosphorylated and di-Gly-modified peptides

Cells were lysed in modified RIPA buffer (50 mM Tris pH 7.5, 150 mM NaCl, 1 mM EDTA, 1% NP-40, 0.1% sodium deoxycholate) supplemented with protease inhibitors (Complete protease inhibitor cocktail tablets, Roche Diagnostics), phosphatase inhibitors, and 5 mM N-ethylmaleimide (Sigma). Lysates were precipitated in acetone and subsequently re-dissolved in denaturation buffer (6 M urea, 2 M thiourea in 10 mM HEPES pH 8.0). Cysteines were reduced with 1 mM dithiothreitol and alkylated with 5.5 mM chloroacetamide (Nielsen *et al*, 2008). Proteins were digested with endoproteinase Lys-C (Wako Chemicals) and sequencing grade modified trypsin (Sigma) after 4-fold dilution in water. Protease digestion was stopped by the addition of trifluoroacetic acid and the precipitates were removed by centrifugation. Peptides were purified using reversed-phase Sep-Pak C18 cartridges (Waters).

The peptides were divided into two portions, one portion (∽5 mg) was used for TiO_2_-based enrichment of phosphorylated peptides and the remaining peptide portion (∽20 mg) was used for antibody-based affinity enrichment of ubiquitylated (di-Gly-lysine containing) and tyrosine-phosphorylated peptides. For antibody-based enrichments, the peptides were taken in immunoprecipitation buffer (10 mM sodium phosphate, 50 mM sodium chloride in 50 mM MOPS pH 7.2) and di-Gly-lysine-modified and tyrosine-phosphorylated peptides were enriched sequentially. First, di-Gly-lysine-modified peptides were enriched with 100 μg of purified di-Gly-lysine-specific GX41 monoclonal antibody (Lucerna) (Xu *et al*, 2010). Subsequently, the flow-through from the di-Gly-lysine affinity enrichments was incubated with 80 μl of pY100 antibody coupled to agarose beads (Cell Signaling Technology) to enrich tyrosine-phosphorylated peptides. Peptides were incubated for 4 h with the respective antibodies at 4°C on a rotation wheel. The beads were washed three times in ice-cold immunoprecipitation buffer followed by three washes in water and elution with 0.15% trifluoroacetic acid in H_2_O.

For the enrichment of phosphorylated peptides, about 5 mg of peptides was dissolved in 50% acetonitrile and TFA was added to a final concentration of 6% (Zhou *et al*, 2013). The peptides were incubated with TiO_2_ microspheres (GL Sciences) and eluted with 25% ammonia. Before MS analysis, the peptides were separated into six fractions using micro-column-based strong cation exchange chromatography (SCX) and desalted on reversed-phase C18 StageTips, as described previously (Rappsilber *et al*, 2007; Wisniewski *et al*, 2009; Weinert *et al*, 2013).

### MS analysis

Peptides were analyzed on a quadrupole Orbitrap mass spectrometer (Q-Exactive, Thermo Scientific) equipped with a nanoflow HPLC system (Thermo Scientific), as described (Michalski *et al*, 2011; Kelstrup *et al*, 2012). Peptides were loaded onto C18 reversed-phase columns (15 cm length, 75 μm inner diameter) and eluted with a linear gradient from 8 to 40% acetonitrile containing 0.5% acetic acid. The mass spectrometer was operated in a data-dependent mode, automatically switching between MS and MS2 acquisition. Survey full-scan MS spectra (m/z 300–1700) were acquired in the Orbitrap. Typically, the 10 most intense ions were sequentially isolated and fragmented by higher-energy C-trap dissociation (HCD) (Olsen *et al*, 2007). Peptides with unassigned charge states, as well as with charge state < +2, were excluded from fragmentation. For di-Gly-enriched samples, charge states < 3 were excluded from fragmentation. Fragment spectra were acquired in the Orbitrap mass analyzer.

### Peptide identification and computational analysis

The raw MS data files were analyzed using MaxQuant (version 1.3.9.21) (Cox & Mann, 2008). Parent ion and MS2 spectra were searched against human or murine proteome databases obtained from the UniProtKB released in February 2012 using Andromeda search engine (Cox *et al*, 2011). Tandem mass spectra were searched with a mass tolerance of 6 ppm for precursor ions and 20 ppm for fragment ions; strict trypsin specificity and allowing up to two missed cleavage sites. Cysteine carbamidomethylation was searched as a fixed modification, whereas N-terminal protein acetylation; methionine oxidation; n-ethylmaleimide modification of cysteines; di-Gly-lysine; and phosphorylation of serine, threonine, and tyrosine were searched as variable modifications. Di-Gly-modified lysines were required to be located internally in the peptide sequence. Site localization probabilities were determined by MaxQuant using the PTM scoring algorithm (Olsen *et al*, 2006; Cox & Mann, 2008). The dataset was filtered based on posterior error probability (PEP) to arrive at a false discovery rate of below 1% estimated using a target-decoy approach (Elias & Gygi, 2007). Statistical analysis was performed using the R software environment. Gene Ontology enrichment analysis was performed using the DAVID bioinformatics resource (Huang *et al*, 2009a,b). GProX was used for clustering phosphorylation sites based on their dynamic regulation (Rigbolt *et al*, 2011). MiMI plugin for Cytoscape was used to create and visualize protein interaction networks (Cline *et al*, 2007; Gao *et al*, 2009). For pathway analysis, GPML file of B-cell receptor pathway was downloaded from NetSlim database (Kandasamy *et al*, 2010) and was modified in Cytoscape for visualization.

### Data analysis for BCR signalosome, phosphoproteome, and ubiquitylome

To define a cutoff for classifying proteins that were significantly enriched in BCR signalosomes, we first separately calculated the median SILAC ratio of proteins quantified in signalosomes isolated after 5 and 15 min of BCR stimulation. We assumed that a majority of the identified proteins in our experiments were bound to beads as background binders, and treated 90% of proteins with the lowest SILAC ratios as background binders. We used the SILAC ratios of these proteins to calculate standard deviation (SD) for SILAC M/L and H/L ratios. To identify BCR signalosome-specific interacting proteins, we required that proteins should have log_2_ SILAC M/L ratio, or log_2_ SILAC H/L ratio greater than median + 2SD. Based on this cutoff, proteins greater than log_2_ SILAC M/L ratio of 1.36 and log_2_ SILAC H/L ratio of 1.86 were considered BCR signalosome-specific interactors. Additionally, we required that these proteins should be enriched ≥ 2-fold in at least two out of four replicate experiments. To classify BCR-regulated phosphorylation and ubiquitylation sites, we used a cutoff of 2-fold change in SILAC ratio, which corresponded to ∽median ± 2.5SD.

### Preparation of retroviruses and generation of HOIP reconstituted cell lines

The generation of A20.2J HOIP^−/−^ and A20.2J HOIP reco cells (HOIP^−/−^ cells reconstituted with full-length HOIP) has been described previously (Hostager *et al*, 2011). To generate A20.2J HOIP^−/−^ cells expressing HOIP ΔRBR, or HOIP Δ379, A20.2J HOIP^−/−^ cells were retrovirally transduced with pMIP-HOIP ΔRBR or pMIP-HOIP Δ379. Forty-eight hours later, cells were treated with 2 μg/ml of puromycin to select the transduced cell population. Retroviral particles were produced in HEK293T cells as described previously (Hostager *et al*, 2011). Briefly, HEK293T cells were co-transfected with pCLeco (Naviaux *et al*, 1996) and pMIP-HOIP constructs. Forty-eight hours later, the cell culture media containing viral supernatant were harvested and used to transduce the target cells.

### Immunoprecipitation and immunoblotting

For immunoprecipitation of BCL10 and FLAG-HOIP, cells were lysed in modified RIPA buffer supplemented with protease and phosphatase inhibitors, and 10 mM N-ethylmaleimide. Cells were incubated with lysis buffer for 15 min at 4°C on a rotating wheel, followed by centrifugation at 13,000 rcf for 20 min. The protein amount in lysates was quantified using Quick Start™ Bradford Protein Assay (Bio-Rad). BCL10 was immunoprecipitated using α-BCL10 antibody (Santa Cruz, #5273) and agarose-conjugated protein G beads (Invitrogen). FLAG-HOIP was immunoprecipitated using agarose-conjugated α-FLAG antibody (Sigma). Immunoprecipitated proteins were separated on SDS–PAGE (4–12% Nu-PAGE gels, Invitrogen). Proteins were transferred onto nitrocellulose membranes, and the membranes were blocked with 5% skimmed milk powder (Sigma) solution in PBS containing 0.1% Tween-20 (Sigma) for 1 h at room temperature. The membranes were incubated with antibodies diluted in 5% BSA for either 1 h at room temperature or overnight at 4°C. The antibodies used for immunoblotting are listed in the Supplementary Table S12. Horseradish peroxidase-conjugated secondary α-mouse or α-rabbit antibodies (Jackson ImmunoResearch Laboratories) and Novex ECL chemiluminescence (Invitrogen) were used for immunodetection.

### *In vitro* protein deubiquitylation

Cells were lysed and ubiquitylated proteins were pulled down using Met1-SUB as described above. After pull-down, the beads were washed twice with ice-cold RIPA with protease and phosphatase inhibitors (but without N-ethylmaleimide) and three times with ice-cold PBS. The beads were resuspended in 30 μl of PBS containing 0.01% Triton X-100 (Sigma) and incubated with 8 μg of purified DUB for 1 h at 30°C. The reaction was stopped by adding 2× LDS sample buffer (Invitrogen) and boiling at 70°C for 12 min. The eluent was subjected to SDS–PAGE and immunoblotting as detailed above.

### Plasmids and site-directed mutagenesis

To generate pcDNA-BCL10, BCL10 encoding cDNA was amplified from pMSCV-FLAG-BCL10 (Addgene plasmid #18718) (Wu & Ashwell, 2008) and cloned in pcDNA3.1+ zeocin vector (Invitrogen). To obtain pcDNA BCL10-LinUBL73P-4X plasmid, cDNA encoding LinUBL73P-4X was synthesized (Geneart service, Invitrogen) and cloned into pcDNA-BCL10 plasmid. pMIP-HOIP ΔRBR and HOIP Δ379 were generated from full-length FLAG-HOIP by deleting the RBR domain, or amino acids C-terminal to 379 of HOIP. To obtain pcDNA-LinUBL73P-4X, the cDNA encoding LinUBL73P-4X was cloned into pcDNA3.1+ zeocin vector. Gateway® entry vectors (pENTR221, Invitrogen) containing ANKRD13A, RAB7A, and RILP cDNA were obtained from the Ultimate™ ORF Clones library (Invitrogen). ANKRD13A and RAB7A cDNAs were shuttled into pcDNA-DEST53 using LR recombinase (Invitrogen), and the cassette containing GFP-ANKRD13A and GFP-RAB7A was then subcloned into the MCS of the pMX-IRES-puromycin vector using the conventional cloning techniques. ANKRD13A mutants (ΔUIM and UIM3/4 mutant) and RAB7A point mutants (S72A, S72D, and S72E) were generated by site-directed mutagenesis in pENTR221 vector, and the mutant cDNAs were transferred into the pMX-IRES-puromycin vector as described above. To obtain FLAG-RILP expressing cDNA, RILP cDNA was shuttled into FLAG tag containing pMX-IRES-puromycin vector. To obtain pcDNA-UBL73P, the point mutation (L73P) was introduced by site-directed mutagenesis in HA-tagged ubiquitin cloned in pcDNA (Addgene plasmid #18712) (Kamitani *et al*, 1997). To generate pcDNA-BCL10-UB2 construct, linear di-ubiquitin (UB2) was PCR-amplified from pcDNA-NEMO-UB2 (Kensche *et al*, 2012) and fused in-frame to BCL10 in pcDNA-BCL10 construct.

### Electroporation and transfection

A20 cells were electroporated with ANKRD13A or ANKRD13A UIM3/4 mutant with Neon® transfection system (Invitrogen) according to manufacturer's protocol. Briefly, 2.5 million A20 cells were resuspended in 100 μl of buffer R1 and mixed with 30 μg of pMX-IRES-puromycin encoding GFP-tagged ANKRD13A WT or UIM3/4 mutant. The following parameters were used for electroporation: 1,350 V, 20 mA, and two pulses. The electroporated cells were cultured for 48 h before using for immunoprecipitation. HeLa or HEK293T cells were transfected using Lipofectamine 2000 (Invitrogen).

### Luciferase assay

HEK293T cells were seeded in a 12-well plate (Thermo Scientific) 24 h before transfection. Cells were transfected with the indicated amount of pcDNA-BCL10, or pcDNA-BCL10–LinUBL73P-4X as well as with 250 ng of pNF-κB Luc (Promega) and 25 ng of pRL-TK Renilla (Promega) for normalizing transfection efficiency. Cells were transfected with Lipofectamine 2000 (Invitrogen) according to the manufacturer's instruction, and 24 h after transfection, luciferase activity was assayed by Dual-Glo® Luciferase Assay System (Promega).

### Immunofluorescence microscopy

HeLa cells were seeded onto poly-d-lysine-coated coverslips (NeuVitro) 48 h before transfection. Cells were co-transfected with GFP-tagged RAB7A and FLAG-RILP using Lipofectamine 2000 (Invitrogen). Forty-eight hours after transfection, coverslips were prepared for immunofluorescence as previously described (Gupta *et al*, 2011). FLAG (Sigma #A2228) and LAMP1 (Abcam #ab24170) antibodies were used to stain FLAG-RILP and the endogenous LAMP1, respectively. α-FLAG was detected using Alexa Flour® 568 goat α-mouse IgG, and α-LAMP1 was detected using Alexa Flour® 647 donkey α-rabbit IgG (Invitrogen). DAPI (DAKO) was used to stain the nucleus. Images were acquired on an inverted epi-fluorescence microscope (Leica DMI 6000B), with 100× oil field objective. For the B-cell microscopy, primary B-cell isolation, retroviral transduction with RAB7A constructs, and imaging were performed as described (Natkanski *et al*, 2013). Mouse B cells were obtained from splenocytes following red blood cell lysis (ACK buffer, GIBCO) and negative selection using anti-CD43 microbeads (Miltenyi Biotec). B cells were stimulated with 1 μg/ml CpG (Sigma) for 24 h and spinfected with retrovirus-containing supernatants. After 48 h, B cells were fixed with paraformaldehyde, permeabilized with 0.1% Tween-20, immunostained for LAMP-1 (Abcam), and followed with staining with a secondary anti-rabbit antibody conjugated with Alexa Flour® 568. 3D epifluorescence images of the GFP and Alexa Flour® 568 channels were acquired and displayed as maximum projections.

### Mass spectrometry data access

The raw MS data for BCR signalosome, phosphorylation, and ubiquitylation analysis have been deposited to the ProteomeXchange Consortium (Vizcaino *et al*, 2014) via the PRIDE partner repository (http://www.ebi.ac.uk/pride/archive/projects/PXD001440) with the dataset identifier PXD001440.

## Supporting Information

Supplementary Table S11

Review Process File
