# Supplementary material for: Systems-wide analysis of BCR signalosomes and downstream phosphorylation and ubiquitylation
Source: Mol Syst Biol. 2015 Jun 2;11(6):810. doi: 10.15252/msb.20145880 (PMC4501846; doi:10.15252/msb.20145880)
Supplement: Supplementary file 1 [file msb0011-0810-sd1.pdf]

Systems-wide analysis of BCR signalosomes and downstream phosphorylation and ubiquitylation

Shankha Satpathy[1,§], Sebastian A. Wagner[1,5,§], Petra Beli[1,6], Rajat Gupta[1], Trine Kristiansen[1,7], Dessislava Malinova[2], Chiara Francavilla[1], Pavel Tolar[2], Gail A. Bishop[3], Bruce S. Hostager[4], Chunaram Choudhary[1,*]

Supplementary figure and table legends

Supplementary Figure 1: Relative quantification of BCR receptor components. The cumulative intensities of BCR components in the signalosomes isolated after 5 and 15 minutes of the receptor activation. The bar chart shows the ratios of cumulative peptide intensities for the indicated BCR components in the signalosomes isolated at 5 (M) and 15 minutes (H). The data are shown for four individual experiments (Exp1- 4).

Supplementary Figure 2: Reproducibility of BCR signalosome, phosphoproteome and ubiquitylome. (**A**) Overlap between BCR signalosome components identified in four experiments. The Venn diagram shows the overlap between proteins that were enriched in BCR signalosomes in four different experiments (Exp1-4). (**B, C**) Correlation between experimental replicates for phosphoproteome and ubiquitylome. Peason correlation coefficient between two independent biological replicates was calculated for the BCR phosphoproteome (**B**) and ubiquitylome (**C**).

Supplementary Figure 3: Regulation of tyrosine phosphorylation in BCR signaling. (**A**) Tyrosine phosphorylation is more robustly upregulated compared to serine and threonine phosphorylation. The significance between the magnitude of up-regulated phosphorylated serine/threonine and tyrosine sites was calculated using Wilcoxon test. The box represents the middle 50% of the distribution and the line within the box represents the median value of the distribution. (**B**) Time-dependent decrease in BCR-induced tyrosine phosphorylation. The line graph shows mean SILAC ratios of tyrosine or serine/threonine phosphorylated peptides after 5 and 15 minutes of BCR stimulation. The mean SILAC ratio of tyrosine phosphorylation showed a decrease between 5 and 15 minutes, whereas there was no

change for serine/threonine phosphorylation. (**C**) A network view of BCR-regulated tyrosine phosphorylated proteins. The size of node reflects the number of phosphorylation sites on each protein and the color indicates log_2 SILAC ratios of proteins with BCR-regulated (up or down) tyrosine phosphorylation.

Supplementary Figure 4: Subcellular localization of GFP-RAB7A WT in Hela cells. Cells were transfected with GFP-tagged RAB7A. Cells were fixed, permeabilized, and stained α-LAMP1 antibodies to detect the endogenous LAMP1.

Supplementary Figure 5: Overlap between proteins identified from TUBE pull-down and di-Gly enrichment . (**A**) The Venn diagram shows the overlap between all proteins that were identified from TUBE pull-down and di-Gly enrichment experiments. (**B**) The Venn diagram shows the overlap of proteins that contained BCR-induced ubiquitylation (SILAC ratio $\geq$2) in TUBE pull-downs and di-Gly enrichment dataset.

Supplementary Figure 6: Enrichment of BCL10 and Met1-UB-specific peptide in TUBE and Met1-SUB pull-downs, and activation of NF-κB by BCL10 and linear ubiquitin fusion protein. (**A**) The enrichment of Met1-UB-specifc peptide (GGMQIVK) in higher molecular weight gel fractions. A20 cells were stimulated with anti-IgG and ubiquitylated proteins were affinity enriched from unstimulated and BCR-stimulated cells using TUBEs (**A**), or Met1-SUB (**B**). Proteins were separated on SDS-PAGE and quantified by SILAC-based mass spectrometry. The table in the panel **A** includes the results from TUBE-based enrichment and shows the number of gel fractions analyzed, the corresponding molecular weight of proteins present in these fractions, and the relative enrichment of Met1-UB-specific peptide in BCR-stimulated cells compared to unstimulated cells, in individual gel fractions. The table in the panel **B** contains the results from Met1-SUB pull-downs and shows the number of gel fractions analyzed, the corresponding molecular weight of proteins present in these fraction, and the relative enrichment of Met1-UB-specific peptide and BCL10 in BCR-stimulated cells compared to unstimulated cells, in each gel fraction. The SILAC ratio indicates the median of quantified peptides. (**C**) Expression of BCL10 and BCL10-LinUBL73P-4X in HEK293T cells. HEK293T cells were transfected with plasmids encoding BCL10 or BCL10-LinUBL73P-4X. Cell lysates were immunobloted against BCL10. (**D**) Activation of NF-κB reporter activity by BCL10 and BCL10-UB2

fusion proteins. HEK293T cells were co-transfected with increasing amounts (0.5µg, 1 µg, or 2 µg) of BCL10, or BCL10-UB_2 construct together with pNF-κB Luc and pRL-TK Renilla. NF-κB transcriptional activity was measured 24 hours later using Dual-Glo® Luciferase Assay System (Promega). Error bars indicate mean ±SEM of 2 independent experiments. Statistical significance was determined by two-tailed Student´s t-test.

Supplementary Table S1: A list of proteins quantified in BCR signalosome analysis (SILAC L= unstimulated, SILAC M = 5 minutes BCR stimulation, SILAC H =15 minutes BCR stimulation).

Supplementary Table S2: A list of all phosphorylation sites quantified after BCR stimulation (SILAC L= unstimulated, SILAC M = 5 minutes BCR stimulation, SILAC H =15 minutes BCR stimulation).

Supplementary Table S3: A list of protein kinases and ubiquitin ligases that were phosphorylated after BCR stimulation (SILAC L= unstimulated, SILAC M = 5 minutes BCR stimulation, SILAC H =15 minutes BCR stimulation).

Supplementary Table S4: A list of GOBP terms that were enriched in proteins containing BCR-upregulated phosphorylation sites.

Supplementary Table S5: A list of proteins quantified in the GFP-RAB7A and RAB7A mutant pull-downs.

Supplementary Table S6: A list of all di-Gly modified (ubiquitylation) sites quantified after BCR stimulation (SILAC L= unstimulated, SILAC M = 5 minutes BCR stimulation, SILAC H =15 minutes BCR stimulation).

Supplementary Table S7: A list of all GO terms enriched in BCR-upregulated di-Gly modification dataset.

Supplemental Table S8: A list of BCR signalosome components that were also ubiquitylated in response to BCR stimulation (SILAC L= unstimulated, SILAC M = 5 minutes BCR stimulation, SILAC H =15 minutes BCR stimulation).

Supplemental Table S9: A list of proteins that were concurrently modified with BCR-upregulated phosphorylation and ubiquitylation (SILAC L= unstimulated, SILAC M = 5 minutes BCR stimulation, SILAC H =15 minutes BCR stimulation).

Supplementary Table S10: A list of proteins quantified in TUBE-based pull-downs after BCR stimulation (SILAC L= unstimulated, SILAC M = 5 minutes BCR stimulation, SILAC H =15 minutes BCR stimulation).

Supplementary Table S11: A list of proteins quantified in Met-1 SUB-based pull-downs after BCR stimulation (SILAC L= unstimulated, SILAC M = 5 minutes BCR stimulation, SILAC H =15 minutes BCR stimulation).

Supplemental Table S12. A list of antibodies used in this study.

Supplemental Figure S1

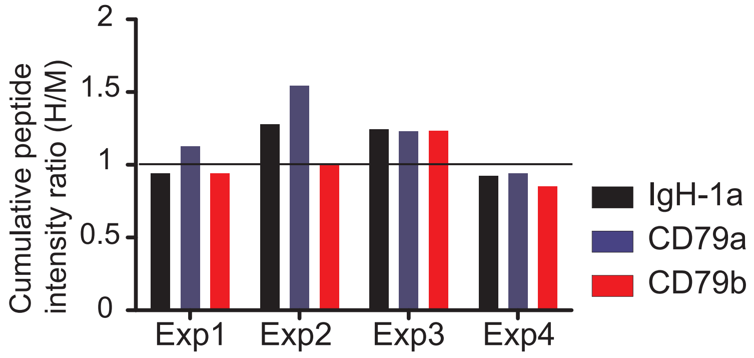

Supplemental Figure S2

A

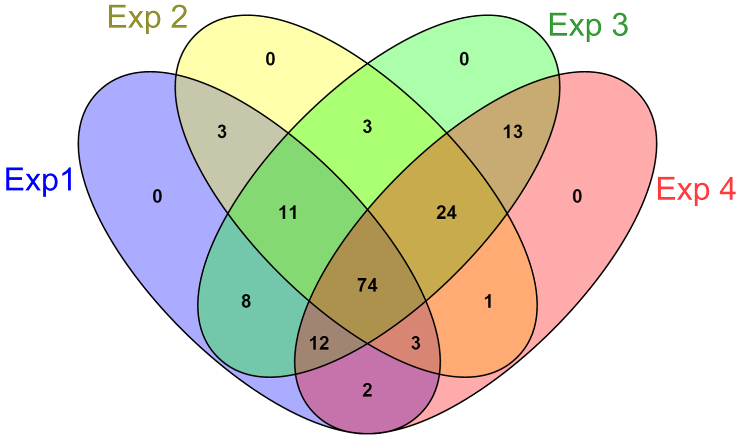

B

BCR phosphoproteome

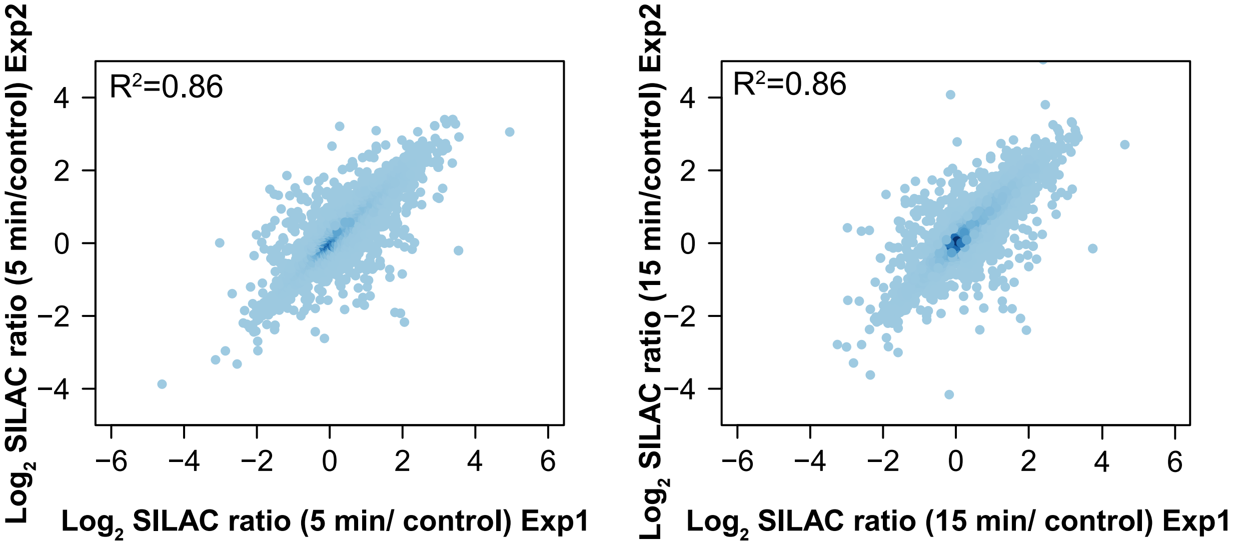

BCR ubiquitylome

C

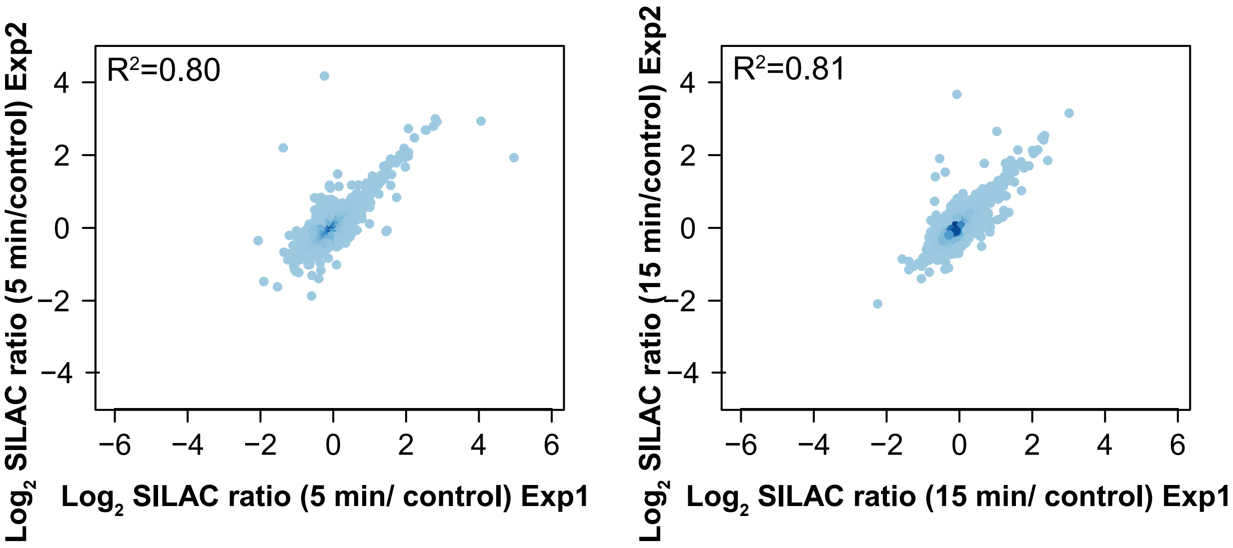

Supplemental Figure S3

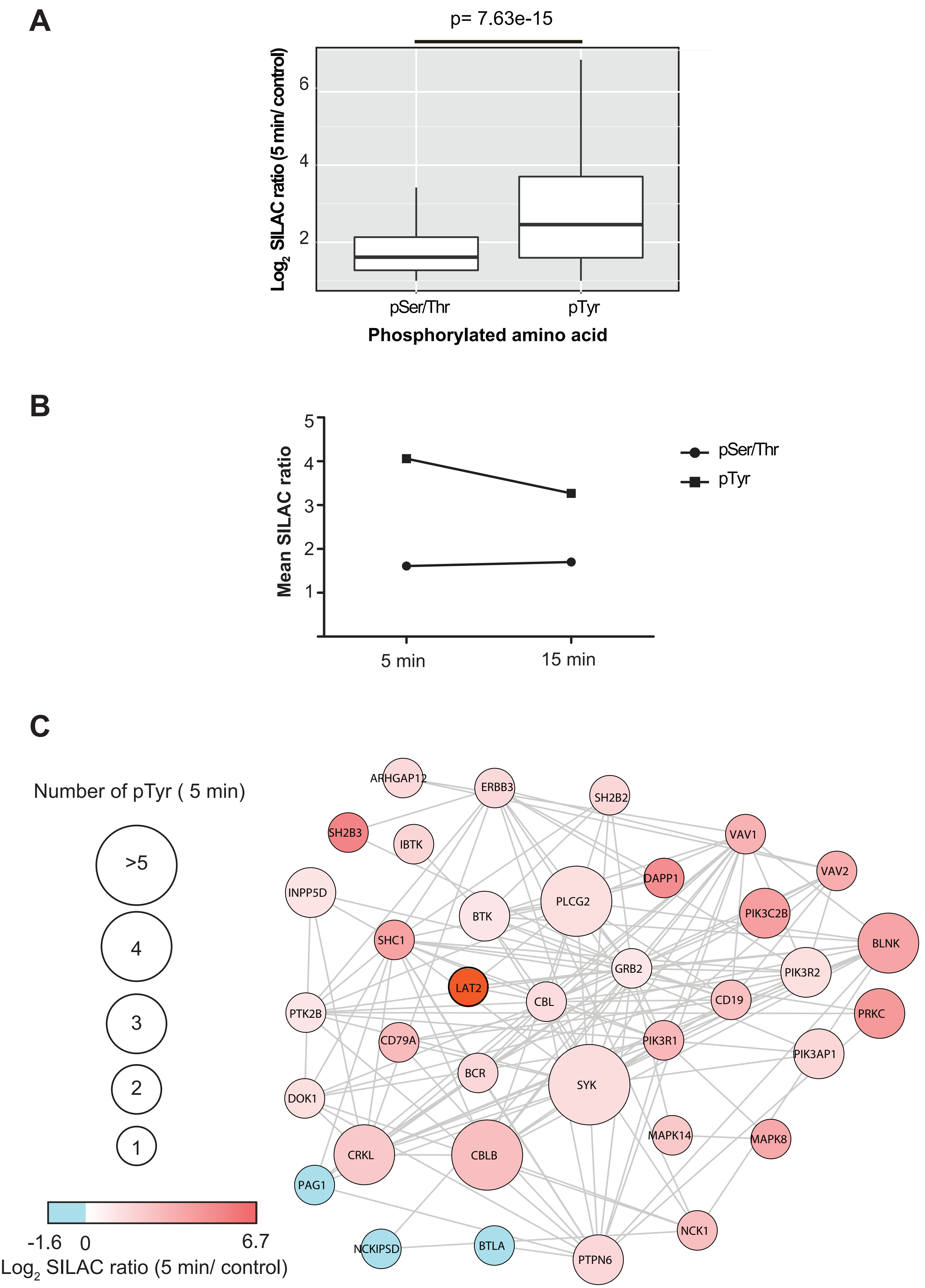

Supplemental Figure S4

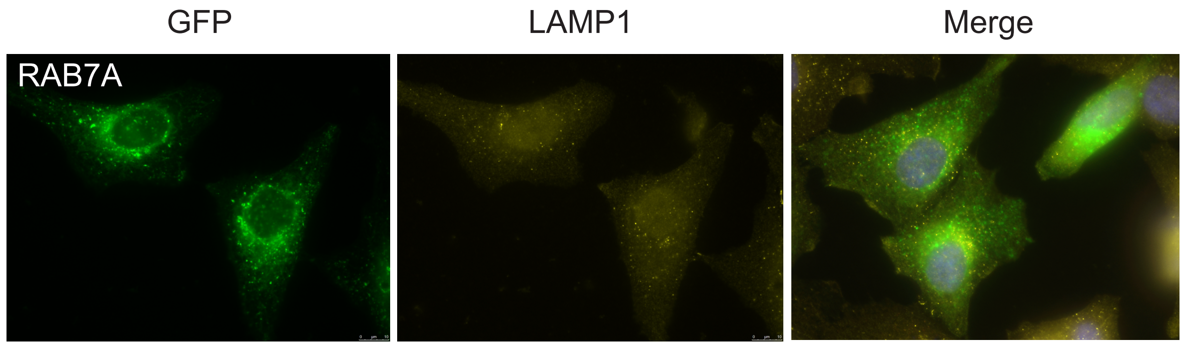

Supplemental Figure 5

A **Number of proteins quantified**

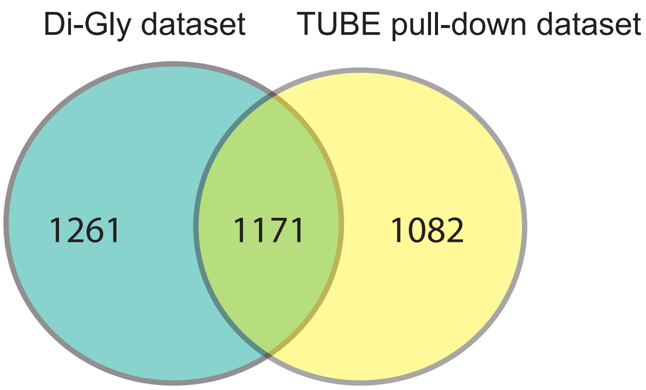

B **Number of proteins with BCR-upregulated ubiquitylation**

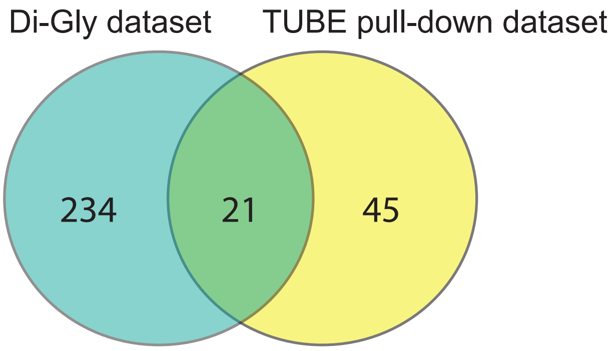

Supplemental Figure S6

A

TUBE pull-down

SDS-PAGE fraction	MW	SILAC ratio: Met1-UB	
		5 min	15 min
1	>110	1.8	2.1
2	~110-70	1.8	2.3
3	~70-53	2.1	2.9
4	~53-40	N/A	N/A
5,6,7	<40	2	1.8

B

Met1-SUB pull-down

SDS-PAGE fraction	MW	SILAC ratio: BCL10		SILAC ratio: Met1-UB	
		5 min	15 min	5 min	15 min
1	>70	4.4	10.2	1.5	2.1
2	~53-70	13.7	8.6	N/A	N/A
3	~30-53	12.1	8.4	3.5	2.9
4	<30	4.7	1.9	N/A	N/A

C

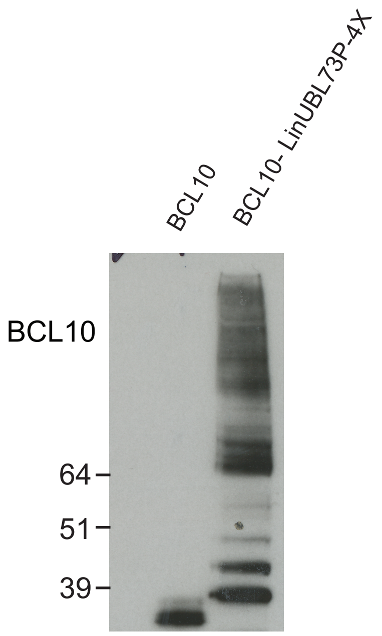

D

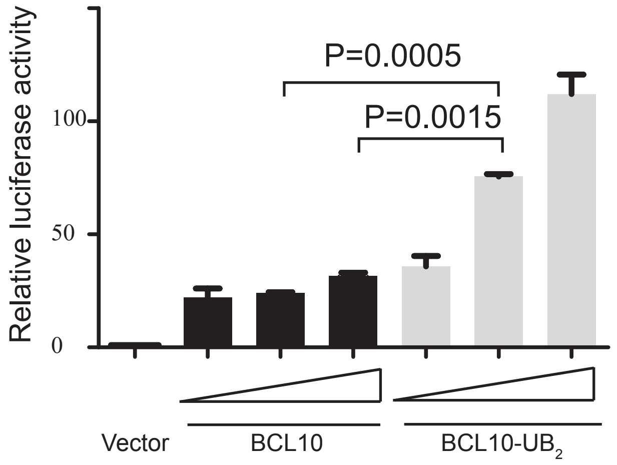

Supplemental Table S12. A list of antibodies used in this study.

Antibody	Catalog no.	Company
**BCL10**	#C78F1, #5273	Cell Signaling Technology, Santa Cruz
**LUB9**	#AB130	Life Sensors
**ACTIN**	#A2228	Sigma
**UBIQUITIN**	#8017	Santa Cruz
**FLAG**	#F1804	Sigma
**IκB**	#9242	Cell Signaling Technology
**p-IκB**	#2859	Cell Signaling Technology
**LAMP1**	#ab24170	Abcam
**BTK**	#3533	Cell Signaling Technology
**p-BTK**	#5082	Cell Signaling Technology
**AKT**	#9272	Cell Signaling Technology
**p-AKT**	#9271	Cell Signaling Technology
**ERK1**	#M7927	Sigma
**p44/42 MAPK (ERK1/2) (T202/Y204)(E10)**	#9106	Cell Signaling Technology
**SYK**	#13198	Cell Signaling Technology
**LYN**	#2732	Cell Signaling Technology
**p85 PI3K**	#4257	Cell Signaling Technology
**ITCH**	#12117	Cell Signaling Technology
