# Supplementary material for: Systems-wide analysis of BCR signalosomes and downstream phosphorylation and ubiquitylation
Source: Mol Syst Biol. 2015 Jun 2;11(6):810. doi: 10.15252/msb.20145880 (PMC4501846; doi:10.15252/msb.20145880)
Supplement: Supplementary file 13 [file msb0011-0810-sd13.pdf]

molecular systems biology

Systems-wide analysis of BCR signalosomes and downstream phosphorylation and ubiquitylation

Shankha Satpathy, Sebastian A. Wagner, Petra Beli, Rajat Gupta, Trine Kristiansen, Chiara Francavilla, Pavel Tolar, Gail A. Bishop, Bruce S. Hostager, Chunaram Choudhary

Corresponding author: Chunaram Choudhary, University of Copenhagen

Review timeline:

Submission date:	29 October 2014
Editorial Decision:	20 November 2014
Revision received:	17 March 2015
Editorial Decision:	15 April 2015
Revision received:	30 April 2015
Accepted:	06 May 2015

Editor: Maria Polychronidou

Transaction Report:

(Note: With the exception of the correction of typographical or spelling errors that could be a source of ambiguity, letters and reports are not edited. The original formatting of letters and referee reports may not be reflected in this compilation.)

1st Editorial Decision 20 November 2014

Thank you again for submitting your work to Molecular Systems Biology. We have now heard back from the three referees who agreed to evaluate your manuscript. As you will see from the reports below, the referees acknowledge that the study provides potentially interesting findings in the context of BCR signaling. However, they raise a series of concerns, which should be carefully addressed in a revision of the manuscript. The referees' recommendations are clear in this regard, so there is no need to repeat their comments listed below.

Reviewer #1:

B lymphocytes play inevitable roles in the adaptive immune responses by recognizing foreign antigens and producing antibodies recognizing those foreign antigens after differentiating into antibody producing cells. Each B cell has one kind of B cell antigen receptor (BCR) and signaling via BCR plays central roles in the development and functions of B cells. Involvement of posttranslational modifications (PTMs) including phosphorylation and ubiquitylation in BCR signaling has been well documented. However, detailed picture of PTMs in BCR signaling has not been well addressed. In this manuscript, Satpathy et al. analyzed phosphorylation and ubiquitylation of proteins elicited by BCR stimulation using multiple proteomic approaches. They found novel protein phosphorylation and ubiquitylation in BCR signaling and dissected their roles. Especially, they found involvement of linear ubiquitin chains, which are specifically generated by the LUBAC ubiquitin ligases, in IgG stimulation in A20 and A20.2J mouse B cell lines. The results presented in this manuscript are convincing and well dissected. Thus the reviewer feels that this manuscript will be accepted in Molecular Systems Biology.

Minor comment.
Page 5 line 15, SYK s not a Src family kinase. It belongs to ZAP70/SYK family.

Reviewer #2:

This work describes identification of the B cell receptor (BCR) signalosome components and characterization of their posttranslational modifications (phosphorylation and ubiquitylation) using SILAC and affinity purification approaches with mass spectrometry analysis. Several follow-up experiments demonstrate biological relevance of some of the identified BCR signaling components, such as a newly identified association of ANKRD13A with the complex, regulation of RAB7A phosphorylation-dependent localization, and BCL10 ubiquitylation. Overall, this study provides interesting insights into the complexity of BCR signaling and diversity of the involved biological processes. However, several concerns should be addressed prior to publication:

Major concerns:
1. For affinity purification experiments following BCR activation (Fig. 1B), the authors analyze stimulation-specific signalosome components, but do not seem to incorporate a control that would isolate unstimulated receptor. Therefore, it is not clear if the components observed at 5min are already present without the stimulus or truly recruited following stimulation. The authors should clarify the experimental details for their control at the zero time point, and should revise their interpretation accordingly.

2. The evidence for the ANKRD13A interaction with the ubiquitylated components of BCR signalosome is insufficient. Deletion of UIMs can potentially lead to disruption of non-ubiquitin specific protein-protein interactions. Additional experiments are required to conclude that ANKRD13A interacts with ubiquitinated components of BCR complex, such as pulldown of wt and mutant ANKRD13A and assessment of the presence of ubiquitylated interacting partners.

3. Fig. 3B: authors should explain the rational for selecting 3 clusters in their BCR-upregulated phosphoproteome. For example, gap statistics can be used to estimate the optimal number of clusters (Tibshirani et al, J. R. Statist. Soc., 2001). In Fig. 3B, cluster 2 includes a large proportion of phosphopeptides with dramatically different kinetics, e.g. phosphopeptides with low membership value (green line).

4. The importance of BCL10 ubiquitylation in NFkB signaling has been shown previously in the context of T cell receptor activation (e.g., Wu&Ashwell 2008 referenced in this manuscript). The reference to this work is only in the methods section. This manuscript would benefit from additional discussion, indicating the commonalities and differences between these studies in B cells and T cells. In particular, this would be helpful when discussing the results showing activation of NFkB reporter in HEK293 cells upon overexpression of ubiquitylated BCL10.

5. It is necessary to explain why the R2 value for the 15min experiment is smaller than for the rest of the experiments (R2=.55, FigS1A); the authors refer to these correlations as significant, which does not seem to be the case for this time point. The reproducibility of the results is important, especially if considering these datasets as resources for the scientific community.

Specific concerns:
1. SILAC labeling efficiency has to be demonstrated.

2. The authors should provide information and discussion regarding the amount of BCR being isolated at 5min and 15min upon IgG stimulation, as this may also impact on the identification of additional 137 interacting proteins at 15min time point (Fig. 2A).

3. For all of the experiments, it is necessary to indicate how the 2-fold SILAC cutoffs were chosen to be considered significant. Specifically, for the experiment in Fig. 1B, significance for SILAC experiments during affinity purification can be assessed by looking at the standard deviation of fold-

© European Molecular Biology Organization

enrichment (see Vermeulen et al., Curr Opin Biotechnol, 2008).

4. PTM enrichment strategy would benefit from some minor clarification. The schematic shown in Fig. 1A implies that the indicated PTMs were enriched sequentially, i.e. pTyr peptides were enriched following the Di-Gly peptide enrichment, etc. Fig.1 legend also calls this strategy a "sequential enrichment." However, the methods section describes three separate enrichment experiments, without a clear mention of whether the flow-through from one enrichment was used for the next enrichment or the samples were split for the different enrichments.

5. Fig. 3C: It is not clear whether all identified significant GO terms are included in the figure or how the cutoff was chosen for these representative GO terms. In addition, a discussion should be included on why these particular processes were activated with different kinetics.

6. Fig 4C: a lysosome marker (e.g., LAMP1) should be included in the images to indicate RAB7A co-localization with the lysosomes.

7. Fig. 5B: same question as for Fig. 3C, how were the enriched GO terms selected?

8. It would help to include a comparison showing overlap between peptides/proteins identified in TUBE and di-Gly IPs to indicate how these two approaches complement each other.

9. Fig. 6C: it would be helpful to add p-value for the K63-linked ubiquitin levels between 5min and 15min time points; it is not clear whether this time-dependent increase is significant.

10. Fig. 7 A and B: it is not clear which bands correspond to ubiquitylated BCL10, LUB9, etc.

11. MW markers should be present on all WBs.

The following typographical errors should be checked:
- p. 4: "... identify proteins that dynamic components of BCR signalosome..."
- p.5: "...were enriched at both the time points..."
- Fig 2D: remove "(min)" and specify the time of pulldown in the figure legend.
- Supp. Table S9 is labeled as Supp. Table S8 in the Excel file.

Reviewer #3:

In this study Satpathy et al. applied comprehensive quantitative mass spectrometry-based proteomics to analyze the dynamic composition of BCR signalosomes and post-translational modifications (phosphorylation and ubiquitylation) of signalosome components as well as downstream effectors not physically associated with the signalosome. All experiments were carried out in A20 cells and quantitative data was obtained using the SILAC method. In order to monitor the dynamic composition of BCR complexes in a stimulation-dependent manner BCR was crosslinked with biotin-labelled anti-IgG F(ab')2 and affinity-purified signalosomes were subjected to LC-MS/MS analysis. In addition to known signalosome components several new proteins were identified, such as the Kelch domain containing proteins KLHDC2 and KLHL6, SPRED1, CUL2, NEDD8 and ANKRD13A. With regard to BCR-dependent phosphorylation events the authors quantified more than 10.000 sites with high confidence, >95 % of which were Ser/Thr sites. Several of the modified proteins have not been implicated in BCR signaling previously: E3 ubiquitin ligase HECTD1, deubiquitylases USP15 and OTUD4, and ZC3HAV1. The authors also detected a strong increase in the phosphorylation of RAB7A in BCR stimulated cells and provide evidence that phosphorylation of RAB7A inhibited its endosomal localization. Finally, to analyze the BCR-associated ubiquitylome the authors first applied the di-Gly capture method to enrich ubiquitylated peptides. They quantified > 6.000 ubiquitylation sites after BCR stimulation, among them multiple RAS isoforms, GNAI2 and STAT3. In addition, the authors used recombinant tandem ubiquitin

binding entities (TUBEs) as a complementary method to isolate ubiquitinated proteins. They observed a significant increase in linear ubiquitination upon BCR crosslinking and showed that Bcl10 is a major substrate of BCR-induced linear ubiquitylation (using a Met1-specific ubiquitin binder). Finally, evidence is provided showing that linear ubiquitylation of BCL10, which requires both HOIP and TRAF6 upstream of HOIP, plays a functional role in the activation of NF-kB signaling in response to BCR activation.

By combining large-scale quantitative analysis of three different aspects of signal transduction, namely signalosome composition, phosphorylation and ubiquitylation of effector proteins, the authors provide a very comprehensive and detailed picture of the signaling dynamics triggered by B cell receptor activation. The data do not provide any unexpected conceptual changes in the understanding of BCR signaling, however they do significantly refine and complete the picture in a quantitative and temporal manner. The authors identified several new proteins as well as PTMs that might play important roles in BCR signaling and for some of them (such as ANKRD13A, RAB7A and BCL10) they provide initial indications of their potential functions. The provided data are mostly of high quality and there are only few points that need to be addressed.

Major comments:
1. Fig. 1B and Fig. 2: As the signalosome is purified using the biotin-labeled anti-IgG F(ab')2 which also serves to stimulate BCR signaling one wonders how BCR signalosome was purified from unstimulated cells? The authors might want to add a respective description (at least in the materials&methods section).

2. The authors concentrate on proteins that associate with the BCR signalosome upon stimulation. However, they do not discuss whether there are proteins that dissociate from the BCR complex upon crosslinking.

3. Fig. 6C: The authors show the abundance of different Ub linkage types isolated using TUBEs after BCR stimulation. The high abundance of linear chains is striking. It markedly exceeds both K48 and K63-linked chains in particular at 15 min of activation. Can the authors comment on this? Do TUBEs have the same affinity for all linkage types under the conditions of the experiment?

4. The authors have quantified more than 6000 Ub sites using the di-Gly method but do not give any number for the TUBE method. Do the proteins mentioned in Fig. 6B comprise all candidates identified with TUBE? The authors might want to provide a more detailed comparison of both methods: number of proteins/Ub sites identified with each method, how many proteins appear in both datasets, limitations of the respective methods etc....

5. Fig. 7C/D. The activities of Otulin and AMSH on both Bcl10 and total ubiquitinated proteins appear very different in the two experiments. While in 7C AMSH removes only a minor part of the ubiquitin modification it has a much stronger effect in 7D (and vice versa for Otulin). In addition a significant Ub signal remains in the sample with AMSH+Otulin.

6. Fig. 8A: HOIP associates with Bcl-10 upon BCR activation. After 10 min the complex is almost completely dissociated again. However, in Fig. 6C (total linear ubiquitination) and 7A (linear ubiquitination of Bcl10) the authors show linear ubiquitination increased markedly till at least 15 min of activation. How can these data be reconciled?

Minor comment:
1. p. 14: reference not cited correctly: (Hostager, 2011 #25)

1st Revision - authors' response 17 March 2015

© European Molecular Biology Organization

Response to reviewers´ comments

We thank the reviewers for the evaluation of our manuscript, and for their helpful comments which have greatly helped in further improving the manuscript. Below, we provide a point-by-point response to the reviewers´ comments.

Reviewer #1:

B lymphocytes play inevitable roles in the adaptive immune responses by recognizing foreign antigens and producing antibodies recognizing those foreign antigens after differentiating into antibody producing cells. Each B cell has one kind of B cell antigen receptor (BCR) and signaling via BCR plays central roles in the development and functions of B cells. Involvement of posttranslational modifications (PTMs) including phosphorylation and ubiquitylation in BCR signaling has been well documented. However, detailed picture of PTMs in BCR signaling has not been well addressed. In this manuscript, Satpathy et al. analyzed phosphorylation and ubiquitylation of proteins elicited by BCR stimulation using multiple proteomic approaches. They found novel protein phosphorylation and ubiquitylation in BCR signaling and dissected their roles. Especially, they found involvement of linear ubiquitin chains, which are specifically generated by the LUBAC ubiquitin ligases, in IgG stimulation in A20 and A20.2J mouse B cell lines. The results presented in this manuscript are convincing and well dissected. Thus the reviewer feels that this manuscript will be accepted in Molecular Systems Biology.

We thank the reviewer for reviewing the manuscript, and for his/her encouraging remarks.

Minor comment.
Page 5 line 15, SYK is not a Src family kinase. It belongs to ZAP70/SYK family.

We apologize for this error. The text has been revised.

Reviewer #2:

This work describes identification of the B cell receptor (BCR) signalosome components and characterization of their posttranslational modifications (phosphorylation and ubiquitylation) using SILAC and affinity purification approaches with mass spectrometry analysis. Several follow-up experiments demonstrate biological relevance of some of the identified BCR signaling components, such as a newly identified association of ANKRD13A with the complex, regulation of RAB7A phosphorylation-dependent localization, and BCL10 ubiquitylation. Overall, this study provides interesting insights into the complexity of BCR signaling and diversity of the involved biological processes. However, several concerns should be addressed prior to publication:

We thank the reviewer for the evaluation of our manuscript, and for his/her thoughtful suggestions.

Major concerns:
1. For affinity purification experiments following BCR activation (Fig. 1B), the authors analyze stimulation-specific signalosome components, but do not seem to incorporate a control that would isolate unstimulated receptor. Therefore, it is not clear if the components observed at 5min are already present without the stimulus or truly recruited following stimulation. The authors should clarify the experimental details for their control at the zero time point, and should revise their interpretation accordingly.

The reviewer correctly points out that our experimental design did not include a control that would isolate unstimulated BCR. Following the reviewer´s suggestion, we have modified Fig 1A, and the text was also revised as following:

Results section:
"To identify proteins that are present in BCR signalosomes, we crosslinked BCRs with biotin-labeled α-IgG F(ab')$_2$ and the associated protein complexes (signalosomes) were affinity purified using streptavidin-conjugated agarose beads (**Fig 1B**). In parallel, control pull-downs were performed by incubating the beads with lysates from unstimulated A20 cells to identify proteins that nonspecifically associated with the beads."

Discussion section:
"We demonstrate that affinity purification of ligand-bound receptors provides an elegant approach to investigate the receptor-associated signaling complexes. However, we were unable to isolate nonactivated BCRs and thus we could not distinguish between constitutively interacting proteins from the stimulus-dependent interactors. For the same reason, it was not feasible to identify proteins that dissociate after BCR activation."

We also wish to mention that we have attempted to include unstimulated BCR pull-down as a control, however, we noted that as soon as we added anti-IgG to cells it resulted in the activation of BCRs, making it difficult to isolate unstimulated BCR from cells. We also tried to add anti-IgG in lysates after cell lysis, but here it was difficult to control the amount of anti-IgG that was retrieved from unstimulated and stimulated cells. This is because when anti-IgG is added to cultured cells only anti-IgG that was bound to BCRs was isolated and the vast majority of anti-IgG that was present in the culture media was excluded from the enrichment. However, when IgG was added in lysates most of the available anti-IgG (that was bound to BCRs as well as unbound) bound to the beads, which distorts the dynamic range in mass spectrometer and hampers accurate comparative analysis of BCR signalosomes from unstimulated and stimulated cells. Because of these reasons we were unable to include unstimulated BCRs as control in our experiments.

2. The evidence for the ANKRD13A interaction with the ubiquitylated components of BCR signalosome is insufficient. Deletion of UIMs can potentially lead to disruption of non-ubiquitin specific protein-protein interactions. Additional experiments are required to conclude that ANKRD13A interacts with ubiquitinated components of BCR complex, such as pulldown of wt and mutant ANKRD13A and assessment of the presence of ubiquitylated interacting partners.

Previously, it has been shown that ANKRD13A interacts with K63-linked ubiquitin, and UIM3/4 are important for this interaction (Tanno et al, 2012, Mol Biol Cell. 2012). To address the reviewer´s concern, we introduced point mutations within ANKRD13A UIM3/4. ANKRD13A UIM3/4 mutant showed reduced binding to BCR signalosomes, suggesting that these UIMs are involved in the binding of ANKRD13A to the ubiquitylated BCR signalosome components (Figure 2E). While our results are consistent with the previous study and suggest a role of UIMs in binding of ANKRD13A to BCR signalosome, we cannot entirely rule out possible contribution of UIMs in ubiquitylation-independent interactions, and this is now acknowledged in the revised manuscript by stating:
"ANKRD13A UIM3/4 mutant showed a reduced binding to BCR signalosome compared to ANKRD13A WT (Figure 2E). These results are consistent with the previously published study (Tanno et al, 2012) and suggest a role of UIM3/4 in the binding of ANKRD13A to ubiquitylated proteins, however, further work is required to delineate the detailed mechanisms of ANKRD13A recruitment to BCR signalosomes."

3. Fig. 3B: authors should explain the rational for selecting 3 clusters in their BCR-upregulated phosphoproteome. For example, gap statistics can be used to estimate the optimal number of clusters (Tibshirani et al, J. R. Statist. Soc., 2001). In Fig. 3B, cluster 2 includes a large proportion of phosphopeptides with dramatically different kinetics, e.g. phosphopeptides with low membership value (green line).

Following the reviewer´s suggestion we have performed gap statistics, which showed that 5 clusters can represent the dynamics of BCR-regulated phosphorylation data. However, we note that many of the GOBP terms enriched in these clusters are shared, implying that the temporal resolution of our analysis is not sufficient to clearly reveal distinct biological processes that are activated at these early time points. Therefore, we have repeated GOBP term enrichment analysis by using all proteins with BCR-induced phosphorylation (Fig 3B).

4. The importance of BCL10 ubiquitylation in NFkB signaling has been shown previously in the context of T cell receptor activation (e.g., Wu&Ashwell 2008 referenced in this manuscript). The reference to this work is only in the methods section. This manuscript would benefit from additional discussion, indicating the commonalities and differences between these studies in B cells and T cells. In particular, this would be helpful when discussing the results showing activation of NFkB reporter in HEK293 cells upon overexpression of ubiquitylated BCL10.

Following the reviewer´s suggestion, we have expanded the discussion by adding the following text to the revised manuscript:
"It is also worth mentioning that the CBM complex is also involved in the activation of NF-κB signaling by TCR and several natural killer (NK) cell receptors, such as NK1.1, Ly49D, Ly49H, and NKG2D (Gross et al, 2008; Marion et al, 2012; Thome et al, 2010). It has been shown that BCL10 is modified with K63-linked ubiquitylation after TCR activation, and this induces the binding of NEMO to the modified BCL10 (Wu & Ashwell, 2008). In the light of our observation that BCR activation induces linear as well as K63-linked ubiquitylation of BCL10, and that NEMO binds to linear, and to lesser extent K63-linked ubiquitylation (Rahighi et al, 2009), it would be interesting to investigate whether linear ubiquitylation is also involved in the activation of NF-κB by TCRs."

5. It is necessary to explain why the R2 value for the 15min experiment is smaller than for the rest of the experiments (R2=.55, FigS1A); the authors refer to these correlations as significant, which does not seem to be the case for this time point. The reproducibility of the results is important, especially if considering these datasets as resources for the scientific community.

We agree with the reviewer that these correlation values do not appear impressive. However, we note that in many interaction experiments the overall correction is modest, even though specific interactions appear consistent in replicate experiments. When the number of specific interactors among all identified proteins is small, as in our case, the overall correlation is not very informative as it is heavily influenced by the ratios of a much larger number of background binding proteins.

Regardless, to improve the quality of our interaction dataset, we have performed two additional replicate experiments for BCR signalosome analyses. Based on these data, we now only consider BCR signalosome components if they had a SILAC ratio that is greater than the median+2SD, and if they were reproducibly identified with SILAC ratio ≥2 in at least 2 replicate experiments (Fig 2A, 2B, and Supplementary Table S1). Eighty percent of BCR signalosome specific proteins were reproducibly identified in at least 3 replicates, and about half of the interactors were identified in all four experiments (Fig S2A). These results suggest a good overall reproducibility of our analyses.

Specific concerns:
1. SILAC labeling efficiency has to be demonstrated.

Our SILAC incorporation tests showed ~97-98% enrichment of the isotope labeled arginine and lysine, and this is now stated in the methods section.

2. The authors should provide information and discussion regarding the amount of BCR being isolated at 5min and 15min upon IgG stimulation, as this may also impact on the identification of additional 137 interacting proteins at 15min time point (Fig. 2A).

To estimate the relative amounts of BCRs in signalosomes isolated after 5 and 15 minutes, we compared the cumulative intensities of peptides corresponding to BCR components (Igh-1a, CD79a, and CD79b) in the signalosomes. In these measurements we did not notice a systematic difference in the relative abundance of these BCR components in signalosome isolated at 5 and 15 minutes (Supplemental Fig S1).

3. For all of the experiments, it is necessary to indicate how the 2-fold SILAC cutoffs were chosen to be considered significant. Specifically, for the experiment in Fig. 1B, significance for SILAC experiments during affinity purification can be assessed by looking at the standard deviation of fold-enrichment (see Vermeulen et al., Curr Opin Biotechnol, 2008).

We have used the following parameters to determine a significance cutoff for BCR signalosome, phosphoproteome and ubiquitylome. For the phosphoproteome and ubiquitylome data we used median±2.5SD (98% confidence) as cutoff, and this corresponded to ~2-fold change in SILAC ratios. For identifying BCR signalosome specific interactors, median±2SD (95% confidence) was used as cutoff. Additionally, we required that BCR signalosome-specific interactors must have a SILAC ratio ≥2 in at least two biological replicates out of four experiments. This is now indicated in the manuscript text (p 5, paragraph 1) and methods (p 24-25).

4. PTM enrichment strategy would benefit from some minor clarification. The schematic shown in Fig. 1A implies that the indicated PTMs were enriched sequentially, i.e. pTyr peptides were enriched following the Di-Gly peptide enrichment, etc. Fig.1 legend also calls this strategy a "sequential enrichment." However, the methods section describes three separate enrichment experiments, without a clear mention of whether the flow-through from one enrichment was used for the next enrichment or the samples were split for the different enrichments.

We apologies to the reviewer for this ambiguity. We have revised Fig. 1A, and methods to clarify the experimental strategy (p23, paragraph 1).

5. Fig. 3C: It is not clear whether all identified significant GO terms are included in the figure or how the cutoff was chosen for these representative GO terms. In addition, a discussion should be included on why these particular processes were activated with different kinetics.

We have repeated GOBP term enrichment analysis (please see above our response to the reviewer´s major comment #3). Most significantly enriched GOBP terms are shown in the Fig 3B, after manually removing the redundant terms. A full list of GOBP terms enriched in the dataset and the corresponding p values are now provided as supplemental data (Supplementary Table S4).

6. Fig 4C: a lysosome marker (e.g., LAMP1) should be included in the images to indicate RAB7A co-localization with the lysosomes.

We have included the requested control in the revised Fig 4B.

7. Fig. 5B: same question as for Fig. 3C, how were the enriched GO terms selected?

GOBP terms were selected based on their p values, after manually removing the redundant terms. A full list of all GO terms enriched and the corresponding p values are provided in Supplementary Table S7.

8. It would help to include a comparison showing overlap between peptides/proteins identified in TUBE and di-Gly IPs to indicate how these two approaches complement each other.

We have performed the requested analysis, and these data are shown in the Supplementary Fig S5. Also, we have included the following text to discuss the complementarities and limitations of the two methods:
"We used both the di-Gly profiling and TUBE methods to analyze BCR-regulated ubiquitylation as each of these approaches have their own pros and cons. For example, while the di-Gly profiling approach allowed quantifying ubiquitylation in a site-specific manner, this approach is unable to identify sites that are present on peptides that are not easily amenable to shotgun proteomics. Also, this approach is not suited to quantify changes in ubiquitylation that occurs through extension of pre-existing substrate-conjugated ubiquitin molecules. The TUBE-based approach overcomes some of these limitations, but it has its own drawbacks. For example, with this method it is not possible to distinguish between ubiquitylated proteins from nonubiquitylated proteins that are co-purified with ubiquitylated proteins. Also, this method is not suited to quantify changes in ubiquitylation at individual sites on proteins, thus the enrichment is determined by the overall ubiquitylation level of proteins. Thus, combining the di-Gly and TUBE-based approaches could provide complementary information for identifying stimulus-triggered ubiquitylation, as exemplified in this work."

10. Fig. 7 A and B: it is not clear which bands correspond to ubiquitylated BCL10, LUB9, etc.

We have now included molecular weight markers, and clarified which bands corresponds to the indicated proteins.

11. MW markers should be present on all WBs.

Molecular weight markers are now indicated on all WBs.

The following typographical errors should be checked:
We apologize to the reviewer for these errors.

- p. 4: "... identify proteins that dynamic components of BCR signalosome..."
Corrected.

- P. 5: "... was enriched at both the time points..."
Corrected.

- Fig 2D: remove "(min)" and specify the time of pulldown in the figure legend.
Fixed.

- Supp. Table S9 is labeled as Supp. Table S8 in the Excel file.
Supplementary table numbers have been updated.

Reviewer #3:

In this study Satpathy et al. applied comprehensive quantitative mass spectrometry-based proteomics to analyze the dynamic composition of BCR signalosomes and post-translational modifications (phosphorylation and ubiquitylation) of signalosome components as well as downstream effectors not physically associated with the signalosome. All experiments were carried out in A20 cells and quantitative data was obtained using the SILAC method. In order to monitor the dynamic composition of BCR complexes in a stimulation-dependent manner BCR was crosslinked with biotin-labelled anti-IgG F(ab')2 and affinity-purified signalosomes were subjected to LC-MS/MS analysis. In addition to known signalosome components several new proteins were identified, such as the Kelch domain containing proteins KLHDC2 and KLHL6, SPRED1, CUL2, NEDD8 and ANKRD13A. With regard to BCR-dependent phosphorylation events the authors quantified more than 10.000 sites with high confidence, >95 % of which were Ser/Thr
sites. Several of the modified proteins have not been implicated in BCR signaling previously: E3 ubiquitin ligase HECTD1, deubiquitylases USP15 and OTUD4, and ZC3HAV1. The authors also detected a strong increase in the phosphorylation of RAB7A in BCR stimulated cells and provide evidence that phosphorylation of RAB7A inhibited its endosomal localization. Finally, to analyze the BCR-associated ubiquitylome the authors first applied the di-Gly capture method to enrich ubiquitylated peptides. They quantified > 6.000 ubiquitylation sites after BCR stimulation, among them multiple RAS isoforms, GNAI2 and STAT3. In addition, the authors used recombinant tandem ubiquitin binding entities (TUBEs) as a complementary method to isolate ubiquitinated proteins. They observed a significant increase in linear ubiquitination upon BCR crosslinking and showed that Bcl10 is a major substrate of BCR-induced linear ubiquitylation (using a Met1-specific ubiquitin binder). Finally, evidence is provided showing that linear ubiquitylation of BCL10, which requires both HOIP and TRAF6 upstream of HOIP, plays a functional role in the activation of NF-kB signaling in response to BCR activation.

By combining large-scale quantitative analysis of three different aspects of signal transduction, namely signalosome composition, phosphorylation and ubiquitylation of effector proteins, the authors provide a very comprehensive and detailed picture of the signaling dynamics triggered by B cell receptor activation. The data do not provide any

unexpected conceptual changes in the understanding of BCR signaling, however they do significantly refine and complete the picture in a quantitative and temporal manner. The authors identified several new proteins as well as PTMs that might play important roles in BCR signaling and for some of them (such as ANKRD13A, RAB7A and BCL10) they provide initial indications of their potential functions. The provided data are mostly of high quality and there are only few points that need to be addressed.

Major comments:
1. Fig. 1B and Fig. 2: As the signalosome is purified using the biotin-labeled anti-IgG F(ab')2 which also serves to stimulate BCR signaling one wonders how BCR signalosome was purified from unstimulated cells? The authors might want to add a respective description (at least in the materials&methods section).

We thank the reviewer (and reviewer #2) for raising this point. We have now clarified our experimental strategy in the materials and methods section, and revised the main text accordingly. Please see our response to reviewer #2, point 1.

2. The authors concentrate on proteins that associate with the BCR signalosome upon stimulation. However, they do not discuss whether there are proteins that dissociate from the BCR complex upon crosslinking.

As detailed in our response to reviewer #2 (point 1), it was not feasible to isolate unstimulated BCRs. Therefore, it was not possible to confidently identify proteins that dissociate after stimulation of BCRs. We have discussed this limitation in the revised manuscript by adding:
“We demonstrate that affinity purification of ligand-bound receptors provides an elegant approach to investigate the receptor-associated signaling complexes. However, we were unable to isolate nonactivated BCRs and thus we could not distinguish between constitutively interacting proteins from the stimulus-dependent interactors. For the same reason, it was not feasible to identify proteins that dissociate from BCR after the receptor activation.”

3. Fig. 6C: The authors show the abundance of different Ub linkage types isolated using TUBEs after BCR stimulation. The high abundance of linear chains is striking. It markedly exceeds both K48 and K63-linked chains in particular at 15 min of activation. Can the authors comment on this? Do TUBEs have the same affinity for all linkage types under the conditions of the experiment?

We wish to clarify that the abundance of ubiquitin linkage peptides shown in the previous Fig 6C was relative, and not absolute. We fully agree with the reviewer that this comparison was confusing, and we have revised the figure to present our key observation about linear ubiquitylation in BCR, which is a novel finding of our work.

4. The authors have quantified more than 6000 web sites using the di-Gly method, but do not give any number for the TUBE method. Do the proteins mentioned in Fig. 6B comprise all candidates identified with TUBE? The authors might want to provide a more

detailed comparison of both methods: number of proteins/Ub sites identified with each method, how many proteins appear in both datasets, limitations of the respective methods etc....
We thank the reviewer for raising this point. Following his/her suggestion, we have elaborated on this by providing the total number of proteins identified in the TUBE experiments and the number of proteins that overlapped with the di-Gly dataset (Supplementary Fig S5). We also added a paragraph in the discussion section to mention the complementarities and limitations of these methods (please see our response to reviewer #2, specific comment 8).

5. Fig. 7C/D. The activities of Otulin and AMSH on both Bcl10 and total ubiquitinated proteins appear very different in the two experiments. While in 7C AMSH removes only a minor part of the ubiquitin modification it has a much stronger effect in 7D (and vice versa for Otulin). In addition a significant Ub signal remains in the sample with AMSH+Otulin.
We have repeated this experiment and the new results are shown in Fig. 7C.

6. Fig. 8A: HOIP associates with Bcl-10 upon BCR activation. After 10 min the complex is almost completely dissociated again. However, in Fig. 6C (total linear ubiquitination) and 7A (linear ubiquitination of Bcl10) the authors show linear ubiquitination increased markedly till at least 15 min of activation. How can these data be reconciled?

The reviewer raises an interesting point here. We have rigorously analyzed BCL10 ubiquitylation and consistently observe a time-dependent increase in its linear ubiquitylation. Also, we reproducibly observe the interaction between BCL10 and HOIP at 5 minutes. However, interaction between ubiquitin ligases and their substrates is often transient and difficult to detect and we feel that this may have limited our ability to accurately quantify the dynamics of the interaction between BCL10 and HOIP. Also, BCL10 is rapidly ubiquitylated following BCR activation, which alters its migration pattern on SDS-PAGE. Thus, while we do not have a clear explanation for this discrepancy, we believe that it is possibly due to technical reasons that make it difficult to accurately quantify the transient BCL10 and HOIP interaction.

Minor comment:
1. p. 14: reference not cited correctly: (Hostager, 2011 #25)
We thank the reviewer for pointing this out. We have now corrected this citation.

2nd Editorial Decision 15 April 2015

Thank you again for submitting your work to Molecular Systems Biology. We have now heard back from the referee who was asked to evaluate your manuscript. As you will see below, this referee is satisfied with the modifications made and thinks that the study is suitable for publication.

Before formally accepting the manuscript we would like to ask you to address the following issues:
- Please provide individual high-resolution files for the main figures.
- As you may already know, we systematically perform a data quality check before acceptance. In this case, our data editor has informed us that it was somewhat difficult to assess the quality of the western blot data shown in the main Figures, due to the low resolution of these panels. As such we would appreciate if you could use higher resolution images for the western blots.

Please resubmit your revised manuscript online, **within two weeks** and ideally as soon as possible. If we do not receive the revised manuscript within this time period, the file might be closed and any subsequent resubmission would be treated as a new manuscript. Please use the Manuscript Number (above) in all correspondence.

Reviewer #3:

The authors have successfully responded to all raised issues and the manuscript is now acceptable for publication.
This work will attract significant attention in the field.

2nd Revision - authors' response 30 April 2015

Following your suggestion, we have provided high-resolution individual figures, and included higher resolution images for Western blots shown in our figures.
